# Mechanisms of tumor resistance to immune checkpoint blockade and combination strategies to overcome resistance

**DOI:** 10.3389/fimmu.2022.915094

**Published:** 2022-09-15

**Authors:** Xiaoting Zhou, Yanghong Ni, Xiao Liang, Yi Lin, Biao An, Xiang He, Xia Zhao

**Affiliations:** Department of Gynecology and Obstetrics, Development and Related Disease of Women and Children Key Laboratory of Sichuan Province, Key Laboratory of Birth Defects and Related Diseases of Women and Children, Ministry of Education, West China Second Hospital, Sichuan University, Chengdu, China

**Keywords:** immune checkpoint blockade, combination therapy, T cell response, resistance mechanisms, immunotherapy

## Abstract

Immune checkpoint blockade (ICB) has rapidly transformed the treatment paradigm for various cancer types. Multiple single or combinations of ICB treatments have been approved by the US Food and Drug Administration, providing more options for patients with advanced cancer. However, most patients could not benefit from these immunotherapies due to primary and acquired drug resistance. Thus, a better understanding of the mechanisms of ICB resistance is urgently needed to improve clinical outcomes. Here, we focused on the changes in the biological functions of CD8^+^ T cells to elucidate the underlying resistance mechanisms of ICB therapies and summarized the advanced coping strategies to increase ICB efficacy. Combinational ICB approaches and individualized immunotherapies require further in-depth investigation to facilitate longer-lasting efficacy and a more excellent safety of ICB in a broader range of patients.

## Introduction

The emergence of immune checkpoint blockade (ICB) has brought the oncology field to a new stage, offering renewed hope for patients with advanced cancer. Over the past decades, ICB, as one of the representative cancer immunotherapies, has produced the broadest impact on cancer treatment (1). ICB, including programmed cell death protein 1 (PD-1), programmed cell death ligand 1 (PD-L1), and cytotoxic T lymphocyte antigen 4 (CTLA-4) monoclonal antibodies, have shown antitumor efficacies in multiple advanced solid tumors since the initial approval of CTLA-4 inhibitors for metastatic melanoma in 2011 by the US Food and Drug Administration (FDA) (2). There are currently three main classes of ICB approved by the FDA in the treatment of various solid tumors, including six drugs targeting the programmed cell death protein 1 (PD-1)/programmed cell death ligand 1 (PD-L1) checkpoint (nivolumab, pembrolizumab, cemiplimab, avelumab, durvalumab, atezolizumab), anti-CTLA-4 checkpoint (ipilimumab), and recently approved anti-LAG-3 (relatlimab) (3).

Unfortunately, most patients suffer primary resistance and do not respond to anti-PD-1/PD-L1 treatments. The limited efficacy of anti-PD1/PDL1 may be attributed to a range of mechanisms involving the whole immune response process. The most straightforward reasons for primary resistance are insufficient tumor immunogenicity, poor CD8^+^ T-cell infiltration, and irreversible T-cell exhaustion. Moreover, some patients with the initial response develop resistance or relapse eventually, which is called acquired resistance (2, 4). The mechanisms accounting for either form of resistance are intricate and complex, which have not been fully cleared up yet. Golnaz Morad et al. systematically divided the factors that affect ICB response into host-intrinsic factors, including tumor cells, non-tumor cells, age, gender, obesity, and gut microbiota, and host-extrinsic factors such as environmental exposures, social pressure, and unhealthy lifestyles. According to their discussion, the role of host systemic and environmental factors should be noted in the study of ICB response (5). Similarly, Aldea et al. overviewed the tumor cell–intrinsic mechanisms and stromal mechanisms. Of note, the different locations of metastasis can lead to an opposite response to ICB (6). Bagchi et al. reviewed the mechanism of ICB resistance from primary and acquired resistance perspectives. Most cancer cell–intrinsic factors contribute to the primary resistance, for instance, the expression intensity of ICB biomarkers, tumor mutation burden, and epigenetic variations. However, the mechanisms of acquired resistance are not well understood, and some common mechanisms may be shared by both types of resistance (7). Genetic mutations are common during the process of tumor progression. Kobayashi et al. summarized six signaling pathways related to ICB resistance. Understanding these could provide potential combinational options for immunotherapy and molecular-targeted therapies. In addition, as a consequence of activating oncogenic drivers or in response to external stimuli, alteration in phenotype plasticity is another integral approach exploited by tumor cells to avoid immune surveillance, thus getting resistance to immunotherapy (8, 9). Based on the analysis of a panel of syngeneic melanoma mouse models, a melanocytic plasticity signature was uncovered to predict the response to ICB and the outcome of patients, implicating the core of plasticity in ICB resistance (10). Novel strategies targeting tumor cell plasticity could be beneficial for patients receiving immunotherapy (11).

A mounting number of preclinical and clinical studies are ongoing to reveal the mechanisms underlying immune checkpoint inhibitor resistance and offer abundant clues for potential combined therapeutic strategies (12, 13). Combination strategies, promising to solve the restrictions of anti-PD-1/PD-L1 treatment, include a combination with traditional chemotherapy and radiotherapy, other immune checkpoint inhibitors, CAR T therapy agonists of the costimulatory molecule, antiangiogenic agents, oncogenic pathway–targeted therapy, microbiota-centered interventions, and metabolic and epigenetic regulation (14–19). Overall, the higher response rates elicited by combination regimens are associated with boosting multiple phases in the cancer-immunity cycle.

This review will discuss the mechanisms underlying ICB resistance, focusing on the changes in the biological function of CD8^+^ T cells. We then highlight existing and emerging strategies to overcome resistance to ICB and boost immunotherapy in preclinical and clinical studies.

## Mechanisms of immune checkpoint blockade resistance from the perspective of immune response process

As is well known, CD8^+^ cytotoxic T lymphocytes (CTLs) play a significant role in antitumor immunotherapy because they are directly lethal to cancer cells. The central theme of ICB immunotherapy lies in the generation or reactivation of this population of cells (20). Antitumor immunity can be described briefly as antigen presentation cells (APCs), such as dendritic cells (DCs), internalize and process tumor-associated antigens (TAAs) in peripheral tissue; then, DCs migrate to lymph nodes and present tumor-peptide-major histocompatibility complexes to naïve CD8^+^ T cells (21). Meanwhile, mature DCs provide the second signal to naïve CD8^+^ T cells by upregulating CD80 and CD86. Upon these efficient stimulations, naïve CD8^+^ T cells differentiate into CTLs. Eventually, CTLs infiltrate lesion sites and kill cancer cells (22). Effective immunotherapy depends mainly on CD8^+^ T cells as well as their successful activation (23). Therefore, we focused on the immune response procedures, especially changes in the biological function of CD8^+^ T cells, for a deeper understanding of the mechanisms of immunotherapy resistance in ICB.

**Figure 1:**
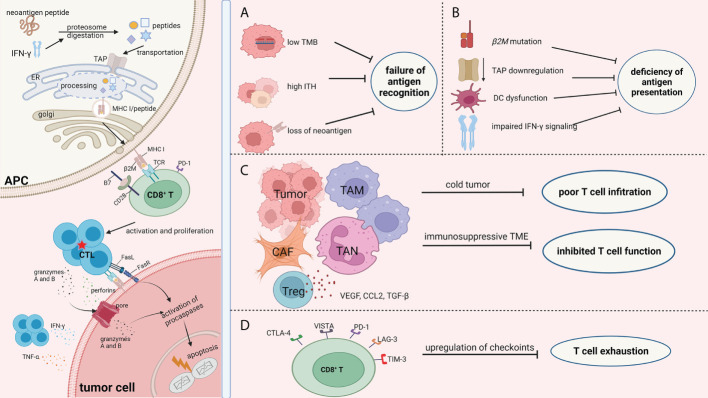
Mechanisms of ICB resistance from the perspective of immune response process. The success of ICB immunotherapy lies in the generation and/or reactivation of the population of CTL cells, which are also the central theme of immunotherapy. The left part of the picture depicts the normal immune response procedure which involves antigen processing and presentation, CD8^+^T cell priming, and the efficient killing of tumor cells by CTLs. Failure of immunotherapy occurs when the different phases of the cancer immunity cycle are compromised and blocked. There are numerous factors that decrease the effect of the antitumor immunity during the fight between tumor cells and immune cells. Regardless of the complexity of the immunotherapy resistance mechanisms, the consequence of these factors can be summarized as **(A)** failure of antigen recognition; **(B)** deficiency of antigen presentation; **(C)** poor CD8^+^ T cells infiltration and inhibited activity of CD8^+^ T cells; and **(D)** exhaustion of CD8^+^ T cells. Therefore, we focused on the immune response procedures, especially changes in biological function of CD8^+^T cells, with an aim to better understand the resistance mechanisms of ICB. The picture was created with BioRender.com. APC, antigen presentation cell; TAP, transporters associated with neoantigen presentation; ER, endoplasmic reticulum; MHC I, major histocompatibility complex class I; TCR, T cell receptor; CTL, cytotoxic T lymphocytes; TMB, tumor mutation burden; ITH, intra-tumor heterogeneity; DC, dendritic cell; TAM, tumor associated macrophages; CAF, cancer associated fibroblasts; TAN, tumor associated neutrophil; CTLA-4, cytotoxic T-lymphocyte antigen 4; VISTA, V-domain Ig suppressor of T cell activation; LAG-3, lymphocyte activation gene‐3; PD-1, programmed cell death protein -1; TIM-3, T-cell immunoglobulin mucin-3.

Drug resistance occurs in blocking the different phases of a cancer immunity cycle, from tumor-specific antigen recognition to presentation, from T-cell activation to recruitment. Overall, the mechanisms of resistance to ICB (**Figure 1**) can be summarized as the (1) failure of antigen recognition; (2) deficiency of antigen presentation; (3) poor CD8^+^ T-cell infiltration; (4) inhibited activity of CD8^+^ T cells; (5) exhaustion of CD8^+^ T cells; and (6) insensitivity to CTL mediated killing.

### Failure of antigen recognition

The immune recognition of tumor cells depends on the HLA-presented antigenic peptide. During cancer progression, gene mutation occurs within cancer cells, resulting in the accumulation of mutated peptides. These neo-peptides are also termed neoantigens because they are different from self-antigens and can be immunogenic most of the time (24). Thus, increased expression of neoantigens within the tumor site can enhance antitumor immunity.

The concept of tumor mutation burden (TMB) has been introduced and utilized as a critical indicator to define tumor antigenicity and evaluate the clinical response to ICB (25). A considerable positive correlation was observed between TMB and the objective remission rate, with a correlation coefficient of 0.7 (26). Non-small lung cancer and melanoma have shown higher TMB and a better response to PD-1 inhibition. Conversely, sarcoma, prostate cancer, and ovarian cancer display lower TMB as well as primary resistance to PD-1inhibition (26, 27). Patients with high TMB (defined as “greater than or equal to 10mut/mb”) were shown to have dramatically higher objective remission rates when treated with pembrolizumab (29%) than patients with low TMB treated with pembrolizumab (6%) in a clinical trial (NCT02628067) (28). On the other hand, tumors with microsatellite instability (MSI) phenotypes, or those with genetic defects in DNA repair enzymes, which is also called DNA mismatch repair deficiency (dMMR), display high mutation loads and more significant response to checkpoint inhibition immunotherapy (29). TMB alone is not a specific determinant of treatment efficacy. Differences in analytical methods, such as different sequencing coverage and depth, lead to differences in sensitivity and specificity when estimating TMB (30, 31). In fact, the durable efficacy of pembrolizumab was still obtained in patients with malignant rhabdoid tumors whose TMB was very low (31). Although high TMB plays a significant role in tumor response to ICB, the prediction of ICB response is far more than TMB estimation.

High intratumor heterogeneity (ITH) can also result in the ineffective recognition of tumor-specific neoantigen and decrease T-cell response to different subclones of tumor cells (32). Pan-cancer analysis indicated that a higher ITH level of tumors was associated with worse survival (33). Wolff et al. demonstrated that low intratumor heterogeneity was a prognosticator of overall survival (OS; p = 0.046) but not TMB (p = 0.16), which suggested that tumors with high ITH were able to escape the immune system despite having high neoantigens (34). McGranahan et al. studied the impact of neoantigen load and neoantigen intratumor heterogeneity on OS in patients who were diagnosed with lung adenocarcinoma (LUAD) and lung squamous cell carcinoma (LUSC). No significant correlation between neoantigen load and neoantigen intratumor heterogeneity with OS in LUSC was discovered, even though the neoantigen burden of LUSC was equally high as LUAD, suggesting the importance of ITH (35).

The loss of neoantigens disturbs the recognition of tumor cells by T cells and causes resistance to ICB. Anagnostou et al. analyzed the data of NSCLC patients who developed required drug resistance after initial response. They discovered 7–18 assumed neoantigens in the resistant tumors. The mechanism of neoantigen loss lies in the deletion of chromosomal regions and the abolition of tumor subclones. The loss of neoantigens was correlated with changes in T-cell receptor clonality (36).

In summary, low TMB and/or high ITH, as well as neoantigen loss, can impact the antigen recognition by CTLs, causing primary or secondary drug resistance to ICBs. In general, tumors with elevated neoantigen expression at the onset of malignant cell cloning will respond better to ICB (37).

### Deficiency of antigen presentation

The activation of CD8^+^ T cells depends on the combination of the T-cell receptor (TCR) and major histocompatibility complex class I (MHC I) molecules (38). MHC I molecule–related neoantigen presentation is modulated by multiple proteins. Beta-2 microglobulin (β2M) is responsible for stabilizing MHC I molecules and promoting antigenic peptide loading (39). The mutations of *β2M* have been found in patients who have acquired resistance to ICBs. For example, in relapse melanoma patients with acquired resistance to pembrolizumab, it was found that a truncating mutation of *β2M* exists in biopsy analysis, leading to the loss of MHC I molecule expression (40). Point mutation, deletion, and the loss of heterozygosity (LOH) were also detected in metastatic melanoma tissues. The degree of *β2M* LOH was tripled in non-responders (approximately 30%) when compared with responders (approximately 10%) and was correlated with inferior OS (41). Apart from melanoma, the links between *β2M* alteration and acquired resistance have been reported in lung cancer (42), gastrointestinal adenocarcinoma (43), and colorectal cancer with a microsatellite instability–high (MSI-H) phenotype (44).

Reduced human leukocyte antigen (HLA) class I gene expression may lead to decreased antigen presentation, thus promoting immune evasion (45). There are up to six different HLA class I alleles in the genome. Highly polymorphic HLA class I genes, including HLA-A, HLA-B, and HLA-C, are responsible for encoding MHC I molecules (46). Eric et al. presented that resistance to KRAS G12D–specific T cell transfer therapy occurred in a patient with metastatic colorectal carcinoma after 9 months. The mechanism of this immunotherapy resistance lies in the deletion of chromosome HLA-C*08:02 in the resistant lesions. Since the existence of the HLA-C*08:02 allele was necessary for KRAS G12D neoantigen presentation and recognition by T cells, its loss directly caused immune evasion (47).

Transporters associated with neoantigen presentation (TAP) are critical players in the MHC I antigen presentation pathway. TAP is a heterodimer consisting of TAP1 and TAP2, both of which are required for peptide translocation (48). The loss or downregulation of TAP in cancers may result in immune evasion and is often associated with an unfavorable prognosis (49, 50). Zhang et al. reported that TAP deficiency resulted in resistance to anti-PD-1, while the efficacy was enhanced in patients lacking both TAP and the non-classical MHC I molecule Qa-1^b^. The results suggested that the immune microenvironment can be altered by inhibiting Qa-1b, especially in the case of defective antigen processing (51). The accumulation of presentation defects may, in turn, lead to a reduced recognition of malignant cells by tumor-specific T cells.

The interruption of IFN-γ signaling, which facilitates MHC I molecule expression on the cell surface in normal conditions, influences neoantigen presentation. Specifically, IFN-γ is an essential signaling molecule for immune-proteasome formation during the degradation of intracellular proteins (52). The loss of IFN-γ signal causes reduced antigen presentation through compromising the coordinated upregulation of the antigen processing procedure (53). Decreased expression of elements in the MHC I antigen presentation pathway can usually be reversed by IFN-γ treatment (53, 54).

The dysfunction of DCs, the most potent antigen-presenting cells, plays a critical role in ICB resistance (55). The deletion of atypical chemokine receptor 4 (ACKR4) in colorectal tumor cells but not stromal cells inhibited the migration of DCs to tumor-draining lymph nodes and impaired antigen presentation. In addition, the knockdown of ACKR4 reduced tumor cells’ sensitivity to ICB (56). High enrichment of myeloid dendritic cells in lung cancer tissues shows an immune activation state, and those patients may benefit from ICB treatment (57). Cytotoxic T-lymphocyte antigen 4 (CTLA4) has a higher affinity to CD80/86 than CD28. CTLA4-positive Treg cells impair the maturation of DCs by binding to CD80/86 and inhibit costimulatory signals (58). Antigen presentation by immature DC or CD80/86 low-expressed DC was unable to stimulate CD8^+^ T cells potently, resulting in CD8^+^ T cells being anergic with low proliferation and insufficient to produce cytokines (59).

### Poor CD8^+^ T-cell infiltration

Different tumor types exhibit various tumor-associated T-cell infiltration densities. The immune landscape of tumors can be divided into three types (1): hot tumor. It is characterized by the enrichment of T cells and their infiltration into tumor tissues, such as lung cancer and melanoma (60) (2). Cold tumor, such as prostate cancer (61) and brain cancer (62), features fewer T cells in the tumor parenchyma or stroma (63). (3) “Immune excluded” tumor. Immune cells do not infiltrate the parenchyma of these tumors, even though there is an abundance of immune cells (64). Compared to hot tumors, the latter two phenotypes rarely respond to ICB immunotherapy, which results in primary drug resistance (65). The infiltration of CD8^+^ T cells into the tumor tissues can be considered a good prognostic parameter for lung cancer and is associated with lymphocyte motility (66).

Genetic alterations within tumor cells have unfavorable effects on T-cell infiltration. *PTEN* loss was associated with reduced T-cell density, lower T-cell expansion, and poor response to PD-1 inhibited therapy in melanoma. Mechanically, the absence of *PTEN* in tumor cells enhances the level of immunosuppressive cytokines, including CCL2 and VEGF, causing less T-cell infiltration and inhibiting autophagy as well, thereby impairing CTL-mediated cell killing (67). *BRAF* mutations are common in melanoma (50%) (68), thyroid papillary cancers (approximately 35%) (69), and colorectal cancers (5%–10%) (70). The biopsy analysis of metastatic melanoma patients showed that selectively inhibiting BRAF with PLX4720 or GSK2118436 induced abundant CD8^+^ T cells in tumors, which provided powerful support for combining BRAF inhibitors with immunotherapy (71). Skoulidis and colleagues showed that *STK11/LKB1* mutation is associated with less expression of PD-L1 and decreased infiltrative CTL density, resulting in primary resistance to PD-1-based immunotherapies in both human and murine *STK11/LKB1*-deficient lung adenocarcinoma (72). Additionally, the loss of *TET2*, which encodes ten-eleven translocation (TET) DNA dioxygenase, is correlated with reduced Th1-type chemokine generation, including CXCL9, CXCL10, and CXCL11, with the downregulated expression of PD-L1 and impaired T-cell attraction to tumor tissues, leading to immune escape and resistance to anti-PD-L1 therapy in the B16-OVA melanoma tumor model (73). NSCLC patients with *EGFR* mutations demonstrated an inadequate response to anti-PD-1 therapy than those with the EGFR wild type. *EGFR* mutation is associated with a reduction in PD-L1 expression, a deficiency in T-cell infiltration, and a decrease in TMB (74).

The elevated vascular endothelial growth factor (VEGF) within the tumor and the consequent aberrant vascular system with high interstitial pressure impair the recruitment of immune cells, correlated with decreased penetration of immune checkpoint inhibitors and increased drug resistance. VEGF inhibits T lymphocyte infiltration within the tumor microenvironment (TME) by suppressing NF-κB signals (75). Tumor-intrinsic STING signaling facilitates *BRCA-1* mutated ovarian cancer cells’ resistance to both PD-L1 and CTLA-4 therapies by upregulating VEGF-A (76). In addition to VEGF, increased C-C motif chemokine ligand 2 (CCL2) was found to be correlated with primary resistance to ICB. CCL2 contributes to insensitivity to ICB by recruiting monocytes and reducing CD8^+^ T-cell infiltration in pancreatic tumors. The poor efficacy of anti-PD-1 therapy can be reversed by CCL2 inhibition or monocyte neutralization (77). Meanwhile, transforming growth factor-beta (TGF-β) produced by cancer-associated fibroblasts (CAFs) was capable of preventing T cells from entering tumor tissue (78). The results from the transcriptional analysis of 298 metastatic urothelial carcinoma samples suggested that the enhanced TGF-β in CAFs was related to poor CD8^+^ T-cell infiltration within tumor parenchyma and weak response to atezolizumab (79). Aside from CAFs, tumor-associated macrophages (TAMs) play an essential role in excluding T-cell infiltration from tumor sites. Interactions between CD8+ T cells and TAMs are durable (at least 20 min), resulting in slowed CD8^+^ T-cell motility (66).

### Inhibited activity of CD8^+^ T cells

**Figure 2:**
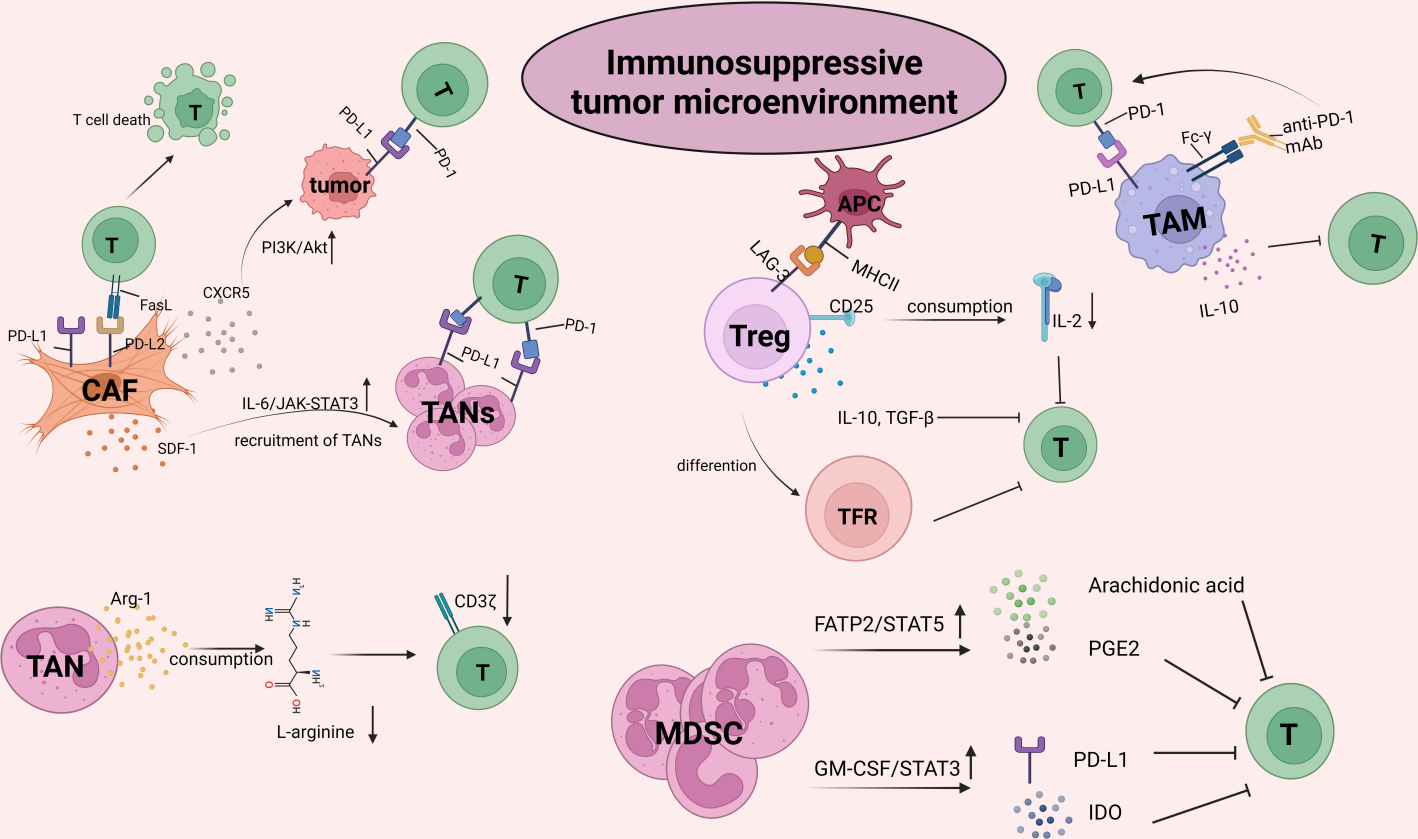
The crosstalk between CD8^+^T cells and the other suppressive cells within tumor microenvironment (TME). TME is infiltrated by different types of innate and adaptive immune cells. The complex crosstalk between these immune cells and tumor cells determines the immune status and the implementation of T cell function, thus to facilitate or inhibit the tumor response to ICBs. With the progression of malignant cells, immune cells within TME, for example, macrophages and neutrophils, are educated into pro-tumor cells. As such, TME becomes progressively immunosuppressive. Immunosuppressive cells inhibit the activity of T cells by upregulating immune checkpoints, capturing anti-PD-1 antibodies and secreting pro-tumor soluble factors such as arg-1, IL-10, TGF-β, promoting tumor immune evasion and resulting in resistance to checkpoint inhibitors. The picture was created with BioRender.com. CAF, cancer associated fibroblasts; TAN, Tumor associated neutrophil; TAM, Tumor associated macrophage; MDSC, myeloid-derived suppressor cell; PGE2, prostaglandin E2; GM-CSF, granulocyte-macrophages colony-stimulating factor; IDO, indoleamine 2,3-dioxygenase; TFR, follicle-regulating T cell.

The TME is infiltrated by diverse innate and adaptive immune cells. The complex crosstalk between immune cells and tumor cells determines the immune status and the implementation of T-cell function, thus facilitating or inhibiting the tumor response to ICB (**Figure 2**). With the progression of tumors, the TME becomes progressively immunosuppressive. Immunosuppressive cells as well as their products facilitate tumor immune evasion and inevitable resistance to checkpoint inhibitors.

Tumor-associated neutrophils (TANs) are one of the critical characteristics of ICB resistance. Immunosuppressive neutrophils from blood and tumors are commonly named granulocyte–myeloid-derived suppressor cells (G-MDSCs) or polymorphonuclear MDSC (PMN-MDSC) (80). Neutrophil-enriched breast tumors display a required resistance to ICB, suggesting a direct suppressive effect on CTLs mediated by TANs (81). In colorectal cancer, the non-response group shows increased levels of MDSC infiltration than the response group treated with anti-PD-1 (82). Consistent with this, a smaller amount of MDSC was found to be linked with a more robust response to ipilimumab in melanoma patients (83). TANs can attenuate the activity of CD8+ T cells by secreting various mediators. One of the essential pathways participating in the immunosuppressive activity of MDSCs is STAT-1-dependent signaling. IFNγ-mediated signals generated by activated T cells can stimulate STAT-1, which subsequently induces the increased expression of immunosuppressive cytokines in MDSCs, such as arginase 1 (Arg-1) (84). Arg-1 results in the downregulation of the CD3ζ chain of T cells by L-arginine exhaustion, suppressing T-cell proliferation and function (85). In addition, the overexpression of fatty acid transporter protein 2 (FATP2) mediated by STAT5 signaling was associated with the enhanced uptake of arachidonic acid and the release of prostaglandin E2 (PGE2) in MDSCs (86). The interaction between tumor cells and MDSCs also plays a critical role in modulating the function of MDSCs. It is reported that MC38 cells secrete the granulocyte–macrophage colony-stimulating factor (GM-CSF) that binds with GM-CSF-R on MDSCs. The combination activates the STAT3 signal within MDSCs, which increases the immunosuppressive effect of MDSC by upregulating indoleamine 2,3-dioxygenase (IDO) and PD-L1, as well as FATP2 (87, 88). The combination of ICB and FATP2 inhibitors delays tumor progression and decreases the expression of PD-L1 on CD8+ T cells (86, 88).

TAMs also significantly contribute to ICB resistance by inducing immunosuppressive interactions within the TME. Notably, TAMs are one of the most enriched immune cells in TME and are involved in both immune stimulation and immunosuppression (89). There are two distinct functional groups of the TAM population, M1 cells (the antitumor macrophages) and M2 cells (the pro-tumor macrophages) (90). Phenotypes can be reversed dynamically between M1 and M2 mediated by cytokines and signals, which is called polarization (91). Firstly, TAMs attenuate T-cell activity by capturing ICB antibodies (mainly of the IgG1 subclass) through Fc-γ receptors, leading to ICB resistance. By using an *in vivo* image to monitor the activity of anti-PD-1 in real time, Arlauckas et al. proved that the anti-PD-1 monoclonal antibody (mAbs) could efficiently bind PD-1^+^ tumor-infiltrating CD8^+^ T cells initially after treatment. Nevertheless, this combination is transient because anti-PD-1 monoclonal antibody are removed by PD-1^-^ TAMs from the T-cell surface within minutes. Measures to block Fc/FcγR binding inhibit the transfer of anti-PD-1 mAbs from CD8^+^ T cells to macrophages *in vivo*, thereby strengthening the therapeutic effect of anti-PD-1 (92). Secondly, TAM reduces ICI efficacy by directly impeding the antitumor capacity of CD8+ T cells. It was found that TAMs directly or indirectly suppress CD8^+^ T cells by secreting IL-10 (93). IL-10 inhibits CD8^+^ T cells primarily by increasing N-glycan branching, thus upregulating the antigenic threshold needed for T-cell activation (94). Thirdly, TAM suppresses T-cell activity by expressing alternative immune checkpoints against ICI efficacy. On one hand, the majority of PD-L1^+^ TAMs are M2 cells, constituting the major TAM population in advanced tumors (95). Thus, high expression of the inhibitory checkpoint on TAMs is inherently a crucial immunosuppressive factor in the TME. On the other hand, PD-L1 expression on TAMs plays a regulatory role during the interplay of TAMs presenting antigenic peptides to homologous effector T cells, which may restrict T-cell superactivation (96).

Under normal conditions, fibroblasts have a low proliferative capacity and metabolic state and are present in a relatively quiescent state in most tissues (97). However, within the TME, tumor cells can promote fibroblast activation by secreting growth factors such as TGFβ, platelet-derived growth factor (PDGF), and fibroblast growth factor (FGF) (98, 99). The CAF-mediated inhibition of T-cell cytotoxic function can be achieved by the upregulation of immune checkpoint molecules. CAFs from melanoma patient biopsies showed the elevated expression of PD-L1 and PD-L2, which directly abrogated CD8+ T-cell function (100). It is suggested that enhanced expression of PD-L2 in CAFs results in antigen-specific T-cell death through PD-L2 and Fas ligand engagement, protecting tumor cells from immune destruction (101). Interestingly, some CAFs also participate in antigen presentation and thus can directly kill activated CD8^+^ T cells *via* the involvement of PD-L2 and Fas ligands (101). PD-L1 and PD-L2 were simultaneously upregulated in CAFs in pancreatic cancer patients. Meanwhile, the CAFs facilitate inhibitory immune checkpoint receptor expression in proliferating T cells. However, the underlying mechanism is not fully understood (102). Apart from upregulating the immune checkpoint directly, CAFs can also indirectly increase the level of immune checkpoint molecules on malignant cells and other cells within the TME. Hepatocellular carcinoma– derived CAFs were demonstrated to recruit neutrophils by secreting SDF1a and facilitating neutrophils’ activation *via* IL-6-JAK-STAT3 signaling. Then, the activated neutrophils upregulated the expression of PD-L1 and exerted a suppressive effect on T-cell immunity (103). CAF-derived CXCL5 is a potent cytokine, which mediates the upregulation of PD-L1 in a PI3K/AKT-dependent pathway within tumor cell lines, including B16, CT26, A375, and HCT116 (104). As such, it is essential to notice that the CAF-mediated dysfunction of CD8^+^ T cells is not limited to a direct interplay of these two cell types.

Regulatory T lymphocytes (Tregs) are of vital importance in tumor progression and their resistance to immunotherapy. Increased infiltration of Tregs has been generally perceived as a biomarker of poor clinical outcomes such as high death hazards and decreased survival (105, 106). Tregs were initially identified as CD4^+^ T cells with increased expression of CD25 (α chain for the IL-2 receptor). FoxP3 was then characterized as a specific marker and major regulator for the maintenance of the immunosuppressive functions of Treg cells (107, 108). Once activated, T cells begin to produce IL-2, which is essential for the sustained proliferation and activation of T cells (109). CD25 has a high affinity to IL-2. Tregs consume IL-2 by upregulating CD25, limiting the sustained activation and proliferation of effector T cells (110). Ren et al. reported that impaired T-cell immunity caused by IL-2 signaling obstruction could be restored by using a low-affinity IL-2 conjugated with anti-PD-1 (PD-1-laIL-2). PD-1-laIL-2, with a higher affinity to PD-1^+^CD8+ T cells than to peripheral Treg cells, was able to amplify the dysfunctional tumor-specific CD8^+^ T cells potently, thus overcoming tumor resistance to ICB (111). Moreover, Tregs suppress T-cell activity by upregulating the expression level of immune checkpoints. Activated Tregs can express lymphocyte activation gene‐3 (LAG-3). CD4^+^CD25^high^Foxp3^+^LAG-3^+^ T cells possess robust inhibitory activity by releasing cytokines, including IL-10 and TGF-β1, without IL-2 (112). It has been proven that Tregs can differentiate into follicle-regulating T (TFR) cells with PD-1 expression, which inhibit the germinal center response (113). TFR cells are distinguished by the coexpression of CXCR5 and GITR2,5 or the transcription factors FOXP3 and BCL-6 (114, 115). TFR cells show advantageous suppressive capacity and *in vivo* persistence compared to conventional regulatory T cells, reducing the effect of an-PD-1 (116). Interestingly, Zappasodi et al. explored the role of a non-conventional subset of CD4^+^FOXP3^-^PD-1^high^ T cells and found that this population of cells expresses a TFR-like phenotype and could limit the functions of the T-cell effector. However, in contrast to regulatory T cells, CD4^+^FOXP3^-^PD-1^high^ T cells were helpful for B-cell activation (117).

### T-cell exhaustion

T-cell exhaustion is characterized by an impaired tumor cell–killing function, the persistent and upregulated expression of inhibitory receptors, and the diverse transcriptional states of normal effector T cells or memory T cells. It is a status of T-cell dysfunction (118). Increased expression of immune checkpoints was reported to be associated with acquired resistance to ICB. *Ntrk1* has been proven to induce the upregulation of PD-L1 in mesenchymal Kras/p53 mutant lung cancer cells by stimulating Jak/Stat signaling, leading to the exhaustion of CD8^+^ T cells within the TME (119). Enhanced expression of T-cell immunoglobulin mucin-3 (Tim-3) was observed in lung cancer patients who progressed after initially responding to anti-PD-1 therapy (120). The coexpression of PD-1 and Tim-3 in T cells was linked with an exhausted phenotype in head and neck squamous cell carcinoma (HNSCC) patients. Mechanically, the upregulated expression level of Tim-3 in T lymphocytes is dependent on the activation of the PI3K/Akt signaling pathway (121). Several checkpoints were coexpressed in TILs isolated from an ovarian tumor mouse model, including PD-1, CTLA-4, and lymphocyte activation gene-3 (LAG-3). The efficacy of single-agent blockade can be impaired by the compensatory enhancement of the other checkpoint molecules, resulting in poor response and resistance (122). With early PD-1 expression and late LAG-3/B- and T-cell lymphocyte attenuator (BTLA) expression, T cells gradually acquire the coexpression of these checkpoint receptors (123). The V-domain Ig suppressor of T-cell activation (VISTA) is another checkpoint of T cells. In melanoma patients with the initial response to anti-PD-1, the density of VISTA-positive T cells was significantly upregulated after treatment, which led to disease progression (124). Increased expression of these inhibitory coreceptors is associated with TCR signaling dysfunction and represents the initiation of negative regulatory signaling, leading to T-cell exhaustion and dysfunction (125). However, exhaustion does not mean the end of T cells’ fate, and their function can be restored by blocking those overexpressed signals mentioned above.

### Insensitivity to cytotoxic T lymphocyte–mediated killing

It is a consensus that CTLs kill tumor cells through two major pathways: granzymes A and B–mediated granule exocytosis and Fas/FasL conjugation–mediated apoptosis induction. Moreover, activated CTLs also secrete cytotoxic cytokines, including interferon-γ (IFN-γ) and tumor necrosis factor-α (TNF-α), to elicit cytotoxicity in tumor cells (126). From this perspective, the sensitive response of tumor cells to cytotoxic factors released by CTLs is vital in preventing immune evasion (127). On one hand, IFN-γ is quite essential for T cells’ penetration into tumors. The effects of antigen-specific immunotherapy depend, to some extent, on tumor sensitivity to IFN-γ (128). The IFN-γ receptor (IFNGR) consists of two subunits, IFNGR1 and IFNGR2. The binding of IFN-γ to its receptor results in the activation of JAK1 and JAK2, which subsequently phosphorylates and dimerizes transcription factor STAT1. STAT1 homodimers then enter the nucleus, binding to specific promoters and initiating the transcription of IFN-γ-regulated genes (129). On the other hand, the release of IFN-γ also mediates the expression of PD-L1 and MHC class I molecules, which may be beneficial for anti-PD-L1 therapy (130).

The dysfunction of the IFN-γ signaling pathway was associated with the primary resistance to ipilimumab therapy in melanoma patients (131). The mutation of JAK1/JAK2 results in PD-L1 depletion and insensitivity to IFN-γ, ultimately causing the primary resistance to anti-PD-1 treatment in melanoma and colorectal cancer patients (40, 132). The depletion of the *IFNGR1* gene in B16 tumor cells suppressed IFN-γ mediated apoptosis and decreased the antitumor effects of anti-CTLA-4 therapy in a mouse model (131). However, the impact of additional IFN-γ pathway genomic alterations other than JAK1 and JAK2 on acquired drug resistance to ICB needs to be further investigated. Of note, the correlations between TNF mutations and survival were not discovered in any type of cancer by Cancer Genome Atlas (TCGA) analysis, indicating that although TNF acts as another cytotoxic factor, its effect is not as sufficient as IFN-γ (133).

## Strategies in overcoming resistance to immune checkpoint blockade: Insights from preclinical cancer models

In accordance with the aforementioned proposed biological mechanisms of non-response to ICB, studies on potential therapeutic strategies addressing resistance mechanisms would be ideal for providing specific insights to improve clinical outcomes. Basically, strategies to reverse ICB tolerance are currently being explored (**Table 1**), which can be outlined as (1) releasing tumor antigens; (2) enhancing antigen presentation; (3) promoting T-cell infiltration; (4) reversing T-cell exhaustion; and (5) CD8+ T-cell stimulation.

**Table 1:** Potential combination strategies to improve the antitumor effect of programmed cell death protein 1 (PD-1)/programmed cell death ligand 1 blockade.

Targeted process	Strategy	Mechanisms	Reference
Releasing tumor antigens	Radiotherapy	Promoting the release of immunogenic neoantigens	(134)
	Chemotherapy	Inducing the ICD	(14)
	Oncolytic viruses	Promoting tumor ICD and “in situ” vaccination	(135)
Enhancing antigen presentation	Histone deacetylase inhibitors	Epigenetically modulating the upregulation of the MHC pathway	(136)
	DNMTi	Elevating the expression of several antigen-presenting molecules	(137)
	STING agonists	Activating cGAS-STING to reverse MHC-I downregulation	(138)
	Polyinosinic:polycytidylic acid (poly I:C)	Inducing MHC I expression *via* NF-κB	(139)
	TLR9 agonists	Augmenting conventional DC (cDC) infiltration to increase antigen delivery	(140)
	Flt3L-poly I:C combined injection	Upregulating the expression levels of CD86, CD40, and MHC II of tumor-infiltrating CD103+ DC	(141)
Promoting T-cell infiltration	PI3K-AKT pathway inhibitors	Promoting T-cell infiltration in PTEN loss melanoma	(142)
Reversing T-cell exhaustion	PORCN inhibitors CGX-1321	Suppressing Wnt/β-catenin signaling to improve CD8^+^ T-cell levels	(143)
	MEK inhibitors	Inhibiting the MAPK signaling pathway to increase T-cell infiltration	(144)
	CDK4/6 inhibitor abemaciclib	Increasing T-cell recruitment with elevated levels of TH1 cytokines/chemokines	(145)
	TGF-β inhibitors	Inducing potent and durable cytotoxic T-cell responses	(146)
	Antiangiogenic therapies	Elevating the expression of adhesion molecules, facilitating the adhesion and extravasation of T cells	(147)
	Low-dose radiotherapy	Reprogramming the TME and inducing T-cell infiltration	(148)
	Mesoporous silica nanoparticle	Eliciting T-cell-recruitment chemokine production and driving CTL infiltration	(149)
	CAR T therapy	Directly providing antigen-sensitive immune infiltration	(150)
	Dual checkpoint inhibitors	Blocking the alternative immune checkpoints to reverse T-cell exhaustion	(151)
	Costimulatory agonists	Reversing T-cell exhaustion and inducing the increase of effector CD8^+^ T cells	(152)
	Targeting transcriptional regulator TOX	Downregulating TOX to ameliorate the exhaustion state of CD8^+^ T cells	(153)
	DNMTi	Epigenetically inducing the rejuvenation of exhausted CD8^+^ T cells	(154)
	Metabolic modulation	Instructing T-cell metabolic programming	(155)
CD8+ T-cell stimulation	Targeting TGF-β	Reducing tumor-infiltrating Tregs	(156)
	CSF1R inhibitors	Inhibiting the differentiation and accumulation of M2-like TAMs	(157)
	Carflzomib	Reprogramming M2 macrophages into the M1-like population through IRE1a-TRAF2-NF-κB signaling	(158)
	NOX4 inhibitors	Reversing TGF-β1-mediated CAF activation	(159)
	Radiotherapy	Increasing CD8^+^ T cells with the reduction of MDSCs and Tregs	(160)
	Microbiota-centered interventions	Regulating the collaboration of microbiota with the TME to promote antitumor T-cell responses	(161)

ICD, immunogenic cell death; MAPK, mitogen-activated protein kinase; DNMTi, DNA methyltransferase inhibitors; NOX4, NADPH oxidase-4.

### Releasing tumor antigens

**Figure 3:**
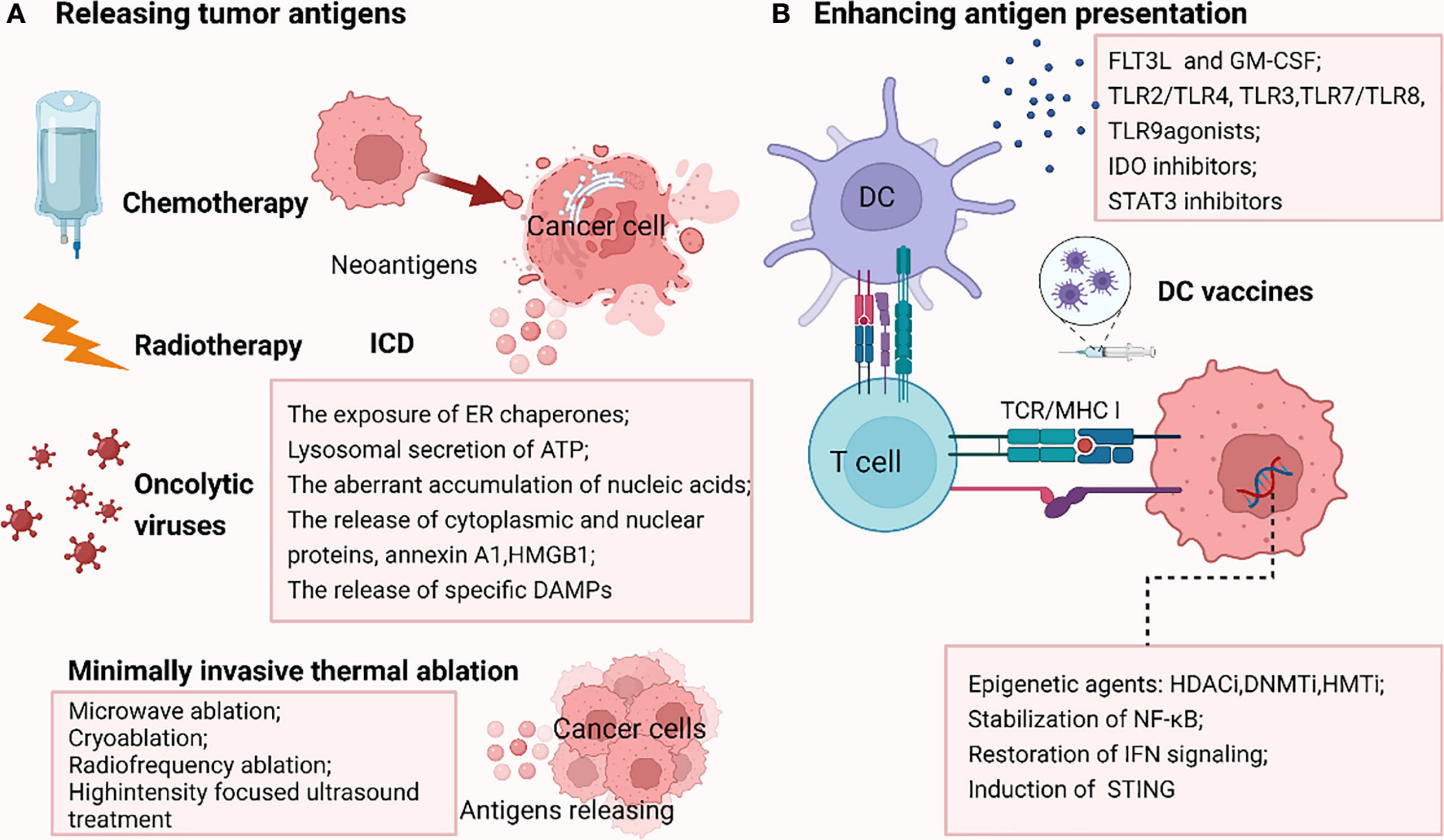
Strategies reversing PD-1/PDL1 blockade by releasing tumor antigens **(A)** and enhancing antigen presentation **(B)**. A.Chemotherapy, radiotherapy and oncolytic viruses could promote the immunogenic cell death (ICD), enhancing the liberation of immunogenic neoantigens, thus increasing the antigenicity in tumors resistant to ICB due to the failure of antigen recognition. In addition, some minimally invasive thermal ablation treatments lead to antigens release as well. **(B)** DNMTi, HDACi, HMTi epigenetically modulate the upregulation of MHC pathway. Stabilization of NF-κB, restoration of IFN signaling and induction of stimulator of interferon genes (STING) also reverse MHC-I downregulation. Besides, stimulation factors including cytokines such as FLT3L (FMS-like tyrosine kinase 3 ligand) and GM-CSF (granulocyte–macrophage colony-stimulating factor), Toll-like receptor (TLR2/TLR4, TLR3, TLR7/TLR8, TLR9) agonists, IDO inhibitors and STAT3 inhibitors could augment the infiltration, activation, and effector function of conventional DCs (cDCs), thus increasing antigen delivery. DC vaccines are also important tools boosting antigen presentation. The picture was created with BioRender.com. ICD, immunogenic cell death; STING, stimulator of interferon genes; FLT3L, FMS-like tyrosine kinase 3 ligand; GM-CSF, granulocyte–macrophage colony-stimulating factor; TLR, Toll-like receptor; IDO, indoleamine- (2,3)-dioxygenase; DC, dendritic cell.

Low TMB and weak or unresponsive neoantigens contribute to the failure of antigen recognition, resulting in ICB resistance. Thus, elevating the release of tumor antigens appears to be a potentially effective approach to reversing ICB resistance (**Figure 3**).

Radiotherapy, as one of the most effective cytotoxic treatments, especially for localized solid cancers, has been considered to cause antitumor immune response apart from causing DNA damage to irradiated cancer cells (15). The abscopal response, originally described in 1953, referring to the shrinkage of tumors outside the irradiated area, has long been thought to involve the mechanisms of the immune response (162). Interestingly, this infrequently occurring abscopal effect could be strengthened by the addition of immunotherapy, which is, in turn, enhanced by radiotherapy (163). Increasing preclinical studies on radiotherapy combined with immunomodulators support the potential role of radiotherapy as an effective immune adjuvant (164). Mechanistically, radiation promotes the release of immunogenic neoantigens, known as TAAs, which play a vital role in *in situ* vaccination (134). Both *in vitro* and *in vivo* studies revealed that the irradiation effectively upregulates cancer testis antigens in the background of necrotic and apoptotic tumor cells and debris, followed with the promotion of the immunological recognition of the tumor (165).

Chemotherapy agents are the conventional treatment for various malignancies. As is known, cytotoxic chemotherapy primarily exerts an antitumor effect by blocking cell division (166). Apart from tumor debulking, chemotherapeutic agents have been demonstrated to promote immunogenic cell death (ICD), which is featured by the exposure of endoplasmic reticulum (ER) chaperones; lysosomal-secreting ATP; the aberrant accumulation of nucleic acids; the release of cytoplasmic and nuclear proteins such as high-mobility group box 1 (HMGB1), annexin A1; and the release of specific damage-associated molecular patterns (DAMPs) (14). Overall, this increasing antigenicity leads to on-target immunostimulatory effects in cancer (167). Recently, a bioresponsive doxorubicin (DOX)-based nanogel has been engineered to directionally release the loaded drugs after being internalized into the TME. These chemoimmunotherapies are promising to conquer the challenges of current ICB-based immunotherapy and provide a paradigm for developing immunomodulatory nanomedicines (168). Data from 12 NSCLC patients suggested that multiple non-mutated neoantigens released from cisplatin-induced apoptotic tumor cells elicited CD8^+^ or CD4^+^ Teff cell responses, which could notably be promoted by anti-PD-1 therapy, correlating with OS (167). Recent trial data on chemotherapy combined with PD-1/L1 inhibitors demonstrate the clinical benefit in patients with NSCLC, triple-negative breast cancer, gastric cancer, and HCC (166, 169, 170).

Oncolytic viruses (OVs) are another selective approach to promoting the release of antigens (171). Similarly, OVs induce tumor ICD and “in situ” vaccination. Subsequently, these soluble TAAs from dying tumor cells facilitate both innate and adaptive antitumor immune responses. Researchers found that in a model of disseminated lung cancer resistant to PD-1 immunotherapy, intratumoral virotherapy elicits CD8^+^ T-cell responses against a set of cancer-specific neoepitopes, overcoming systemic resistance to PD-1 immunotherapy (135). However, different OVs are not capable of inducing ICD equally (172). Thus, incorporating ICD-related DAMP genes seems to be a further attractive option to enhance immunogenicity. In this way, OVs function as engineering platforms for combination immunotherapy. Still, challenges exist in allowing OVs to arrive at the directed primary and metastatic tumor position to perform systematic therapeutic effects (173).

Hopefully, many novel strategies for promoting tumor antigen release are under study. Minimally invasive thermal ablation treatments such as microwave ablation, cryoablation, radiofrequency ablation, or high­intensity focused ultrasound treatment are the common selective therapies for patients with inoperable tumors. Interestingly, these local applications of extreme temperatures lead to the release of antigens from the necrotic tumor lesion, enhancing the activation of the tumor-specific immune response. However, the effect of single thermal ablation is too limited, and appropriate immunomodulators are required for promoting an effective therapeutical systemic antitumor immune response (174–176). Recently, a novel tumor microenvironment ROS/GSH dual-responsive nanoplatform consisting of chemophotodynamic therapy and synergistical control-release PTX has been designed to induce the release of DAMPs after tumor cell pyroptosis, boosting the curative effect of anti-PD-1 treatment in a CT26 tumor model (177).

### Enhancing antigen presentation

The deficiency of antigen presentation represents another major challenge in ICB therapy, which is caused by multiple factors as stated above, including MHC I defects, β2M/HLA gene loss, deficient IFN signaling, and dysfunctional DCs (178). Aiming at these abnormalities is a promising strategy to improve the responsiveness to ICB regimens (**Figure 3**).

The epigenetic control of immune resistance has been implicated as associated with an overall loss of antigen presentation *via* the loss of antigen expression or downregulation of MHC I (179). Histone deacetylases (HDACs) are one class of epigenetic regulators, comprising four families (class I, IIa, IIb, and IV). HDACs appear to have crucial roles in both innate and adaptive immune responses. HDAC1 and HDAC2 have been reported to negatively mediate antigen presentation by inhibiting the main transcriptional regulator of MHC class II genes (180). Accordingly, histone deacetylase inhibitors (HDACis) can epigenetically modulate the upregulation of the MHC pathway, facilitating the immune targeting of cancer cells (136). Four HDACis (e.g., romidepsin, belinostat, vorinostat, and panobinostat) have been approved by the FDA for lymphoma and/or multiple myeloma treatment. In both colon and ovarian cancer cell lines, HDACi treatment promoted increased antigen processing and antigen presentation (181). The efficacy of combining HDACi with PD-1 inhibitors has been evaluated in multiple preclinical cancer models, including melanoma, ovarian cancer, breast cancer, and lung cancer, showing great promise (136, 182, 183). Other epigenetic agents such as DNA methyltransferase inhibitors (DNMTis) as well as histone methyltransferase inhibitors (HMTis) have also been indicated to improve antigen presentation by elevating the expression of several antigen-presenting molecules, thus enhancing the recognition and activation of immune cells (137). Based on these exciting preclinical results, a combination of DNMTi or/and HDACi with ICB has undergone clinical trials in advanced colorectal cancer (NCT02512172), non-small cell lung cancer (NCT01928576, NCT00387465), head and neck cancer (NCT03019003), and gastrointestinal cancers (NCT03812796) (184).

Apart from the epigenetic modification of MHC I antigen presentation, targeting pathways associated with MHC I expression has been described to reverse MHC I downregulation and boost immunotherapy efficacy. Potential therapeutic strategies include the stabilization of NF-κB, restoration of IFN signaling, and induction of stimulator of interferon genes (STING) (138, 139). Notably, the effects of NF-κB and IFNs are pro- or antitumorigenic in different stages and types of tumors. Accordingly, both negative and positive regulators of NF-κB and IFNs have been reported to upregulate MHC I expression (185).

Several strategies to augment conventional DC (cDC) infiltration, activation, or effective function have been proposed to increase antigen delivery and enhance the efficacy of ICB. The stimulation factors include Toll-like receptor (TLR2/TLR4, TLR3, TLR7/TLR8, TLR9) agonists, IDO (indoleamine- (2, 3)-dioxygenase) inhibitors, and STAT3 inhibitor cytokines such as GM-CSF, and FLT3L (FMS-like tyrosine kinase 3 ligand) (186). For example, combining pembrolizumab with a synthetic CpG oligonucleotide TLR9 agonist, SD-101, exhibited greater clinical efficacy than PD-1 blockade alone in a phase Ib trial, which was associated with elevated tumor-infiltrating DC characteristics (140). Similarly, Flt3L-poly I:C combined injection significantly induced the upregulating expression levels of CD86, CD40, and MHC II of tumor-infiltrating CD103^+^ DC and promoted DC immunogenic function, eventually enhancing antitumor responses synergized with anti-PD-L1 Ab treatment in BRAF-mutant and B16 melanoma mouse models (141). Nanomaterials have recently been applied in facilitating the tumor antigen presentation of DCs. A cationic nanoscale metal–organic framework (nMOF) was designed to exert the effects of local immunogenic photodynamic therapy treatment and CpG stimulation, enhancing antigen presentation and synergizing with ICB to induce tumor regression in a breast cancer model (187). Moreover, “next-generation” DC vaccines, essential tools for anticancer therapy, have been suggested to be a desirable combinatorial counterpart for ICB, especially in tumors with low mutational burden (188).

### Promoting T-cell infiltration

**Figure 4:**
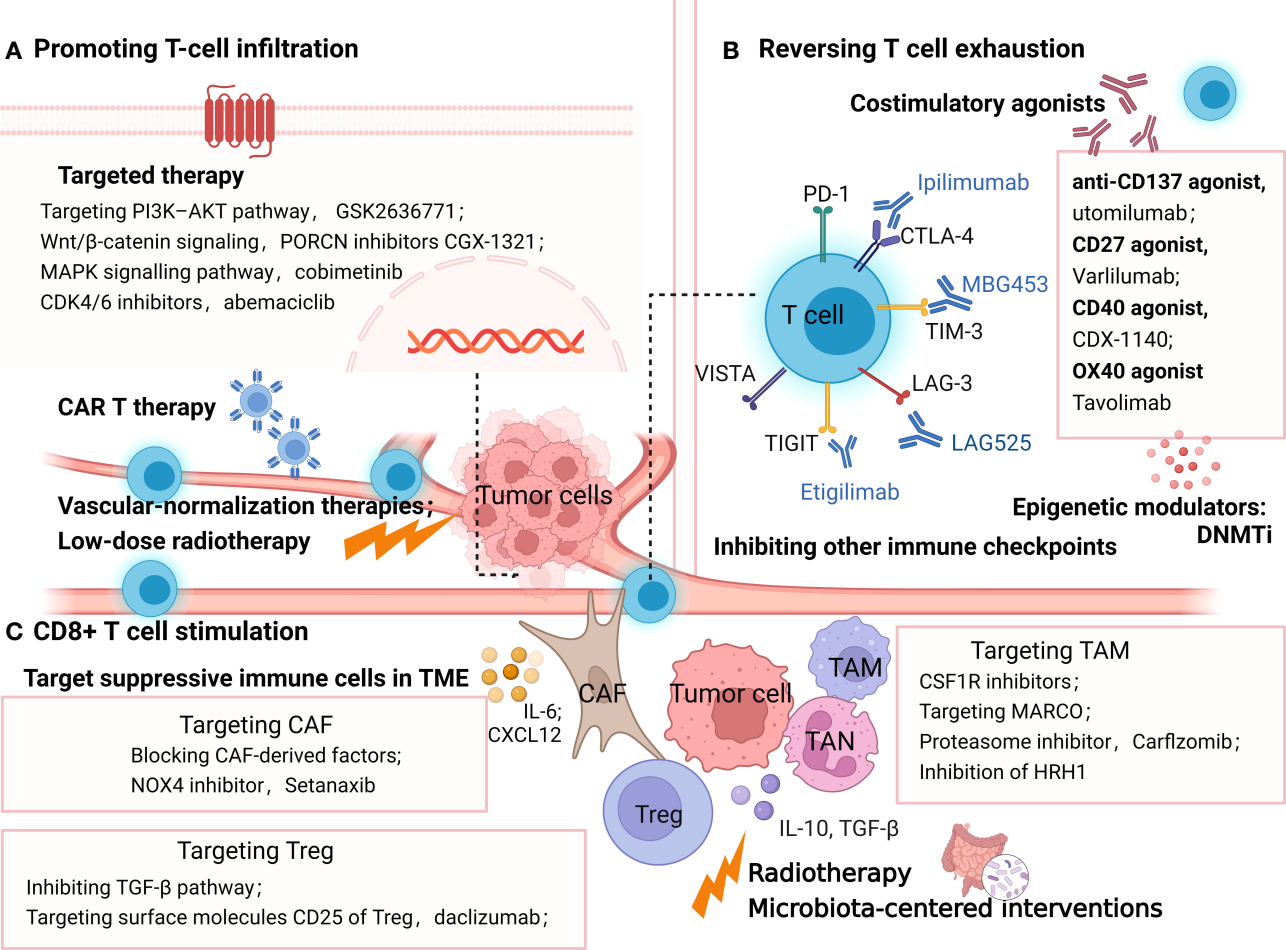
Strategies overcoming resistance to PD-1/PDL1 by promoting T-cell infiltration **(A)**, reversing T cell exhaustion **(B)**, and CD8+ T cell stimulation **(C)**. **(A)** methods promoting T-cell infiltration include targeted therapy, vascular-normalization therapies, CAR T therapy and low-dose radiotherapy; **(B)** treatment options to reinvigorate of T cell exhaustion include blocking the alternative immune checkpoints, targeting co-stimulatory receptors, inhibiting soluble immune suppressive mediators and epigenetically coordinating exhausted CD8+ T (Tex) cells. **(C)** strategies targeting immune-suppressive cells in TME such as TAM, Treg and CAF to stimulate T cells. In addition, radiotherapy and microbiota-centered interventions also reprogram the immunosuppressive TME, promoting antitumor T-cell responses. The picture was created with BioRender.com. CAR, chimeric antigen receptor, Treg, regulatory T lymphocytes; DC, dendritic cell; TAM, tumor associated macrophages; CAF, cancer associated fibroblasts; MARCO, macrophage receptor with collagenous structure; HRH1, histamine and histamine receptor H1.

As a robust prognostic biomarker, tumor-infiltrating lymphocytes are influenced by multiple mechanisms, including genetic alterations within tumor cells, aberrant vasculature, and elevated immunosuppressive factors like TGF-β (12, 146, 189, 190). Low lymphocyte infiltration mainly accounts for the limited efficacy of ICB in many tumors, especially in the immune-infiltrated and -excluded phenotypes (191). Hence, promoting T-cell infiltration *via* targeting these factors provides an outlook on the future for improving ICB effectiveness (**Figure 4**).

mRNA nanoparticles reactivating the tumor suppressor PTEN have been proven to significantly elicit antitumor immune responses and restore the therapeutic effect of ICB in PTEN-null prostate cancer and a PTEN-mutated melanoma model by promoting CD8^+^ T-cell infiltration (190). Furthermore, a drug candidate D18 could suppress the downregulation of PTEN expression by increasing KDM5A abundance, which also potentialized the efficacy of various ICBs in multiple tumor models (192). Moreover, targeting the PI3K-AKT pathway downstream of PTEN is a selective approach to elevate tumor-infiltrating T cells. For example, the PI3Kb inhibitor GSK2636771 sensitized PTEN-null melanomas to both CTLA-4 and PD-1 inhibitors and promoted T-cell infiltration to enhance the antitumor activity *in vivo* (142). Wnt/β-catenin signaling is another tumor-intrinsic pathway associated with poor spontaneous T-cell infiltration. Many inhibitors targeting WNT signaling have been developed to restore T-cell infiltration and reestablish anticancer immunity with ICB. In ovarian cancers, a typical “cold” immune phenotype, PORCN inhibitors CGX-1321 suppressing Wnt/β-catenin signaling, has been confirmed to improve CD8^+^ T-cell levels in the omentum TME (143). Other Wnt signaling inhibitors such as the anti-FZD7 antibody, β-catenin inhibitor DCR-BCAT, DKK1 inhibitor, and WNT inhibitor have been suggested to exert immunomodulatory effects as well (193). Furthermore, clinical trials combining Wnt inhibitor and ICB are ongoing, including DKN-01 (DKK1 antibody) plus pembrolizumab (NCT02013154) and PORCN inhibitor WNT974 combined with spartalizumab (NCT01351103) (194, 195).

The mitogen-activated protein kinase (MAPK) signaling pathway, another oncogenic signaling pathway associated with shaping tumor immunogenicity, has been proposed to be a promising target combined with ICB therapies (12). In a preclinical model of BRAF(V600)-mutated metastatic melanoma, anti­PD1 therapy in combination with BRAF and MEK inhibitors contributed to complete tumor regression with increasing T-cell infiltration into tumors (144). Similarly, it has been reported in colon cancer (the CT26 model) that MEK inhibition promotes the accumulation of TIL by preventing the death of CD8+ T cells triggered by chronic TCR stimulation (196). Clinical studies of MAPK signaling inhibitors plus ICB have shown encouraging results. In BRAF V600–mutated melanoma patients, treatment with the combination of atezolizumab (anti-PD-L1) plus vemurafenib (BRAF inhibitor) + cobimetinib (MEK inhibitor) promoted 71.8% objective responses (a complete response rate of 20%). Meanwhile, the run-in of cobimetinib and vemurafenib contributed to the increase of circulating proliferating CD4+ T-helper cells (197).

Cyclin-dependent kinases 4 and 6 (CDK4/6) inhibition has been highlighted to exert antitumor immune response *via* promoting antigen presentation and enhancing CD8^+^ T-cell infiltration (145). The FDA-approved CDK4/6 inhibitor abemaciclib has shown preclinical synergistic antitumor effects with PD-1 inhibitor in breast cancer mouse models, the ID8 murine ovarian cancer model, and the colon adenocarcinoma murine model, which depends on increased T-cell recruitment with elevated levels of TH1 cytokines/chemokines (198–200).

Immunosuppressive cytokine TGFβ has received growing attention in cancer immunotherapy for its ability to block the antitumor immune response by limiting T- cell infiltration (201). Preclinical models suggested that coinhibiting TGF-β and PD-L1 induced potent and durable cytotoxic T-cell responses, transforming tumors from an excluded to an inflamed phenotype (146, 202). Strategies targeting TGF-β are under development, including the TGF-βRI kinase inhibitor galunisertib, neutralizing antibodies against the mature TGF-β cytokines, antibodies against TGF-βRII, and soluble TGF-β receptor traps, some of which are undergoing clinical trials in combination with anti-PD1 antibodies (203, 204).

As previously described, VEGF-induced immunosuppression inhibits T lymphocyte infiltration in the TME, hampering the therapeutic effect of ICB. In several earlier preclinical studies, vascular-normalization therapies have been proven to facilitate the transformation of the immunosuppressive TME toward an immune-supportive phenotype (205), which manifests as the aggregation of antitumor T cells and DC maturation inside tumors (206). In addition, the process of increased T lymphocyte infiltration induced by antiangiogenic therapies was partly associated with the elevated expression of adhesion molecules (intercellular adhesion molecule–1, vascular cell adhesion molecule-1), which facilitated the adhesion and extravasation of T cells (147). In preclinical mouse models and clinical trials, antiangiogenic agents significantly improved immunotherapy outcomes (205, 207). The various antiangiogenic therapeutic agents mainly consist of anti-VEGFA monoclonal antibodies such as bevacizumab, inhibitors of angiopoietin-2, and VEGFR tyrosine kinase inhibitors (TKIs) such as sorafenib (207). Some of them are presently undergoing clinical trials combining with ICB, receiving more significant clinical benefits than monotherapy in some early data (19).

In addition to the combination of targeted therapies mentioned above, low-dose radiotherapy has been reported to reprogram the TME and induce T-cell infiltration in mouse models of immune-desert tumors (148). Meanwhile, in “inflamed” human tumors, the preexistent intratumoral T cells not only survived radiotherapy but also acquired improved antitumor effects with the increasing production of IFN-γ (208).

It is also noteworthy that biomaterials at the nanoscale have been explored to establish a T-cell-inflamed TME and overcome resistance to ICB. Mesoporous silica nanoparticles were reported to elicit T-cell-recruitment chemokine production and drive CTL infiltration in multiple tumor models resistant to PD-1 antibodies (149). A supramolecular gold nanorod has been reported to reprogram the TME and improve TILs, significantly augmenting ICB therapy, which depends on the hyperthermal activation of ICD and genome editing of PD-L1 (209).

Moreover, chimeric antigen receptor (CAR) T cells may be a direct approach to provide antigen-sensitive immune infiltrates, implying a new opportunity for patients with less immunogenic or “noninflamed” tumors. CAR-T therapy could target T cells directly to tumor cells by genetically modifying T cells (210). Since the initial proposition of CAR-T in 1989, its antitumor efficacy and persistence have been improved due to altering the construction in the advanced generations of CAR-T. Based on these remarkable clinical responses, the FDA has approved four anti-CD19 CAR T-cell products and one anti-BCMA CAR T-cell therapy in different hematological cancers (211). However, the clinical efficacy of CAR T cells in the solid tumor has shown much less satisfactory results. One of the major obstacles includes the fact that PD-1-mediated immunosuppression leads to the poor persistence and dysfunctions of CAR T cells (150). Therefore, ICB and CAR T-cell combination therapy holds promise to refresh the immune system and enhance therapeutic efficacy. A synergy effect has been reported in the combination of PD-1 blockade and CAR-T cell therapy (212). In a transgenic Her-2 recipient mice model, anti-PD-1 antibody combined with CAR T cells showed the enhanced activation and proliferation of anti-Her-2 T cells, with the significant regression of established tumor (213). Other preclinical studies have shown the synergistic antitumor activity of combination therapies in thyroid cancers (214) and pleural mesothelioma (215). Some encouraging clinical results suggested the safety, low toxicity, and clinical responses of combinatorial treatment. One case report demonstrated five patients with diffuse large B-cell lymphoma who endured progression/relapse post-CART19/20 therapy received anti-PD-1 treatment (sintilimab or camrelizumab). Three of five patients had objective responses, including two complete responses and one partial response (216). Similarly, E. A. Chong et al. reported that in 12 B-cell lymphoma patients who were relapsing after or refractory to CD19-directed CAR T-cell therapy, anti-PD1 ICB (pembrolizumab) treatment showed safety and clinical responses (217). Based on these promising preclinical results, a series of one-half of clinical trials exploring the combination immunotherapy of CAR T cells and PD-1 blockade agents for multiple malignancies are under investigation, including relapsed/refractory Hodgkin lymphoma (NCT04134325), classical Hodgkin lymphoma (NCT05352828), relapsed/refractory B-cell lymphoma (NCT04539444), HER2-positive sarcoma (NCT04995003), and glioblastoma (NCT03726515). Some early results of clinical trials suggested the safety and promising efficacy of this combination in patients with malignant pleural disease (218), relapsed/refractory (r/r) diffuse large B-cell lymphoma (219), and relapsed/refractory aggressive B-cell non-Hodgkin lymphoma (220). However, minimal response with no meaningful durability has also been reported in two relapsed, refractory (R/R) B-cell non-Hodgkin lymphoma patients receiving the combination therapy of bispecific CAR T cells and PD-1 inhibitors (221). Therefore, further research is needed to confirm the therapeutic efficacy and optimal administration method of this combination treatment.

### Reversing T-cell exhaustion

As stated above, T-cell exhaustion is characterized by the increased expression of suppressive cytokines and inhibitory receptors, including PD-1, CTLA, LAG-3, TIM-3, VISTA and ITIM domain (TIGIT), hierarchical decreased cytokine production (IL-2, TNF, IFNγ), and reduced proliferative capacity, with underlying distinct epigenetic states (222, 223). Accordingly, upcoming treatment options to overcome ICB resistance by the reinvigoration of T-cell exhaustion (**Figure 4**) include blocking the alternative immune checkpoints, targeting costimulatory receptors, inhibiting soluble immune- suppressive mediators, and epigenetically coordinating exhausted CD8^+^ T (Tex) cells (224–226).

Combining blockade treatments against multiple inhibitory receptors or combining checkpoint inhibitors with costimulatory agonists is a promising way to reinvigorate exhausted CD8^+^ T cells. Desirable therapeutic outcomes have been indicated in the preclinical and clinical studies of many tumors (227). Alternative targeting IRs include anti-TIM-3(MBG453), anti-LAG-3(LAG525), anti-TIGIT (etigilimab), anti-VISTA (JNJ-61,610,588), and anti-B7-H3 (enoblituzumab) (228–231). Accordingly, a wide range of combination strategies are undergoing research in various malignancies both preclinically and clinically. For instance, ipilimumab (anti-CTLA-4) plus nivolumab (anti-PD-1) is the most well-studied immuno-oncology (IO) combination showing comparatively better efficacy in multiple advanced tumors. It has become the earliest dual ICB treatment that received FDA approval in September 2015 for the first-line therapy of metastatic melanoma. Currently, this combination has been approved for the treatment of advanced renal cell carcinoma (RCC), metastatic colorectal cancer with MMR/MSI-H aberrations, PD-L1-positive (≥1%) metastatic NSCLC, and HCC as well. Noteworthily, the increasing incidence and intensity of the adverse events have been reported in the combining blockade, which suggest the importance of further studies (151). Costimulatory agonists are another good choice for reversing T-cell exhaustion in treating ICB. For example, the anti-CD137 agonist utomilumab has been shown to induce the increase of effector CD8+ T cells and improve survival in synergy with ICB in an ovarian cancer model (232). Recently, a growing number of agonist antibodies targeting immune costimulatory receptors are in clinical development for cancer indications, such as CD27 agonist varlilumab (CDX−1127) and CD40 agonist CDX−1140, OX40 agonist tavolimab (MEDI0562). Although none have been approved to date, combination approaches are still full of therapeutic potential (152).

Pauken et al. demonstrated that PD-1 blockade alone minimally remodeled the Tex epigenetic landscape. Hence, epigenetic modifiers, or T-cell epigenomic engineering with checkpoint blockade, may help reacquire durable immune memory against tumors (233). The transcriptional regulator TOX has recently been highlighted to be involved in programming CD8+ T-cell exhaustion transcriptionally and epigenetically, which is associated with plenty of transcription-factor networks downstream of TCR signaling (225). The knockdown of TOX ameliorated the exhaustion state of CD8+ T cells, enhancing the response to ICB treatment in an HCC mouse model (234), suggesting a new strategy to maximize immunotherapeutic efficacy by the downregulation of TOX expression. Interestingly, coblocking PD-1 and TIGIT could reinvigorate TOX-expressing PD-1^high^CD8+ TILs with better therapeutic outcomes in bladder cancer patients (153). Other modulators of the epigenetic landscape stated above, such as DNMTi, have also been found to induce the rejuvenation of exhausted CD8+ T cells, synergizing with a PD-1 inhibitor in a prostate adenocarcinoma mouse model (154).

Metabolic insufficiency play a crucial function in modulating T-cell exhaustion, implicating that metabolic modulation is a selective way to rejuvenate exhausted T cells, eliciting superior antitumor immunity (17, 155). In addition, ICB has been demonstrated to exert an inhibitory effect on immune cells’ metabolism and suppress glycolysis while increasing FAO and lipolysis. Therefore, the combinations of ICB with metabolic interventions appear to be ideal opportunities to improve antitumor effects *via* reversing immune metabolic dysfunctions (235). Many metabolic interventions have been exploited, such as enhancing mitochondrial fitness, enforcing fatty acid oxidation, and ameliorating ER stress (236). For example, in a B16 melanoma mouse model, metformin combined with anti-PD-1 therapy promoted increasing tumor clearance with an elevated intratumoral T-cell function. In addition, this reinvigoration of T cells mediated by metformin is associated with modulating the oxygen tension of the TME (237).

### CD8^+^ T-cell stimulation

Various elements of the TME, including TANs, TAMs, CAFs, and Tregs, play critical immune-suppressive roles in mediating resistance to ICB. Correspondingly, therapies combined with ICB and strategies targeting these immune-suppressive cells appear to overcome resistance and improve clinical outcomes (**Figure 4**).

As is known, Tregs mediate tumor resistance against ICB in multiple ways, including upregulating the expression of other immune checkpoints including LAG-3, TIM-3, GITR, TIGIT, and VISTA; secreting high levels of TGF-β; and increasing the activation of the PI3K signaling pathway (238, 239). In glioblastoma, a typical immunologically ‘cold’ tumor, the suppressive Treg cells were converted toward CD4 effector T cells by an agonistic antibody (αGITR), which promoted the cure rates in GBM models combined with PD1 antibodies (240). Similar results have been reported in the coblockade of PD-1 and other immune checkpoints (241, 242). Importantly, this combined immunotherapy needs to be adapted to the specific immune environment for each tumor type. Targeting TGF-β is another appealing approach to reducing tumor-infiltrating Tregs and improving response to ICB treatment. R. Ravi et al. invented bifunctional antibody–ligand traps (Y-traps), simultaneously inhibiting the TGF-β pathway and CTLA-4 or PD-L1. This engineered antibody (a-CTLA4TGFβRIIecd and a-PDL1-TGFβRIIecd) significantly counteracted Tregs and restored beneficial TH1 cells in the TME, exhibiting superior antitumor efficacy than either the CTLA-4 antibody or PD-L1 antibodies in human melanoma (A375)–bearing NSG mice (156). Other strategies such as daclizumab, targeting the surface molecules CD25 of Treg, have been experimented both preclinically and clinically. Daclizumab administration reprogrammed Tregs. However, it also diminished activated Teff, showing no augmentation of T-cell responses in metastatic melanoma patients (243). Obviously, Treg-silencing strategies coupled with ICB require a deeper investigation of the crosstalk between the TME and Tregs.

As a vital source of PD-1, TAM has been demonstrated to hinder ICB efficacy by capturing ICB antibodies, secreting inhibitory cytokines, and expressing coinhibitory molecules. TAM-centered strategies are promising treatments to improve the efficacy of ICB agents (244, 245). CSF1R inhibitors enhanced the therapeutic efficacy of PD1 blockade by inhibiting the differentiation and accumulation of M2-like TAMs in melanoma models (157). Another monoclonal antibody targeting MARCO (macrophage receptor with collagenous structure) has also been reported to switch the TAM phenotype and boost checkpoint therapy effectively in melanoma tumor–bearing mice, which notably was induced by activating NK-cell-mediated killing other than T- cell-directed immunotherapy (246). Carfilzomib, a proteasome inhibitor approved by the FDA to treat relapsed/refractory multiple myeloma patients, has been supported to reprogram M2 macrophages into an M1-like population through IRE1a-TRAF2-NF-κB signaling and synergize with PD-1 inhibitors to reduce tumor growth in an autochthonous lung cancer model (158). Intriguingly, a recent study revealed that the high expression of histamine and histamine receptor H1 (HRH1) attenuated response to immunotherapies *via* polarizing TAMs toward an M2-like immunosuppressive phenotype. Hence, the HRH1 knockout or inhibition of HRH1 on macrophages with antihistamines reshaped the transcriptomic landscape of immune cells and blocked immune resistance when combined with anti-PD-1 treatment in mammary tumor and colon cancer mice models. In agreement with these results, the clinical data suggested that preexisting allergy or high histamine levels contributed to the inadequate immunotherapy responses in cancer patients (247). The similar antitumor properties of histamine dihydrochloride have been proven in MC-38 colon carcinoma and EL-4 lymphoma mouse model (248). However, in the murine cholangiocarcinoma (CAA) model, TAM blockade by anti-CSF1R failed to reduce CCA growth due to the compensatory infiltration of G-MDSCs. Meanwhile, the dual inhibition of TAMs and G-MDSCs was sufficient to enhance the efficiency of the PD-1 inhibitor in the orthotopic mouse model of CCA. Notably, the response rate to the ICB monotherapy of CAA patients is only 5.8% (249). Thus, targeting these immunosuppressive elements, particularly TAMs, is significant in potentiating PD-1 blockade.

Targeting CAF in the suppressive TME would be another valuable option to improve immunotherapy efficacy. Specifically, the targeted strategies include depleting CAF, interrupting their tumor-promoting ability, blocking CAF activation, and reverting CAF to a quiescent state (250). The inhibition of fibroblast activation protein (FAP)–positive CAF has disappointing results in metastatic colorectal cancer patients, possibly due to off-target effects (251). In recent years, single-cell RNA sequencing has characterized the heterogeneity of CAF in multiple tumor types, which suggests that targeting the subtype of CAF therapy may require a more nuanced approach (252). Blocking CAF-derived factors such as IL-6 and CXCL12 has been demonstrated to increase the accumulation of T cells and boost response to ICB in the models of multiple cancers (253). The ROS-producing enzyme NADPH oxidase-4 (NOX4) inhibition has been demonstrated as a well-studied approach to reversing TGF-β1-mediated CAF activation and promoting the transformation into a quiescent fibroblast-like phenotype (254). Using the NOX inhibitor GKT137831 (setanaxib) with immunotherapy can improve clinical outcomes in CAF-rich solid tumor models, indicating that reversing myofibroblastic CAFs to ‘normalized’ by setanaxib may be a considerable way to resensitize CAF-rich tumors to ICB, such as head and neck, colorectal, esophageal, and pancreatic cancers (255).

Apart from aiming at a specific group of cells or cytokines, radiotherapy is an appealing approach to shifting the immunosuppressive TME in the presence of immunotherapy. Combinatorial therapy has been shown to significantly increase CD8^+^ T cells by reducing MDSCs and Tregs, compared with RT or immunotherapy alone (160, 256). However, the immunosuppression effect of RT was known as well. Those irradiated cells that died of apoptosis could release anti-inflammatory cytokines such as TGF-β and adenosine to reduce tumor tolerance (257). Therefore, the definition of the optimum dose, appropriate fraction, and suitable target site of RT is fundamental (258).

Microbiota-centered interventions have recently gained growing attention for the engagement of the gut microbiome in primary and acquired resistance to ICB in different tumors such as melanoma, RCC, NSCLC, pancreatic ductal adenocarcinoma, and colon cancer (18, 259). Studies have proposed that regulating the collaboration of microbiota with the TME could contribute to metabolic changes, promoting antitumor T-cell responses and ameliorating anti-PD-1 blockade resistance (161). B. Routy et al. revealed that *Akkermansia muciniphila* and *Enterococcus hirae* are the primary factors in eliciting immunological changes, increasing CCR9^+^CXCR3^+^CD4^+^ T lymphocytes, which rely on interleukin-12 (18). Deep mechanisms accounting for the immunomodulatory effects of the gut microbiome remain to be explored. Nevertheless, manipulating the gut ecosystem is a profitable strategy to facilitate a better immune response (260). The specific interventions include supplementation with probiotics, the transfer of the fecal microbial content, microbiome-based metabolite therapy, and the depletion of the unfavorable bacterial taxa by proper oral antibiotics as well as dietary interventions, some of which have been evaluated in early phase clinical studies (261, 262). Intriguingly, researchers found that orally supplementing camu-camu, a polyphenol-rich berry, could circumvent anti-PD-1 resistance by reprogramming the TME in a microbiome-dependent way (263).

## Therapeutic trials to validate resistance mechanisms

### Combining anti− programmed cell death protein 1 (PD-1)/programmed cell death ligand 1 with conventional cytotoxic chemotherapy

Based on the importance of chemotherapy in traditional cancer treatment and the beneficial immunomodulating effects of chemotherapy in the map of PD1/PDL1 therapy, chemotherapy has been the most widely used combination strategy approved in various indications so far and chemoimmunotherapy has become a standard of treatment for some cancer patients. The FDA granted pembrolizumab plus chemotherapy (pemetrexed and platinum) as the first-line therapy for advanced non-squamous NSCLC based on the clinical trial KEYNOTE-021 in 2017. Later in 2018, pembrolizumab plus carboplatin and either paclitaxel or nab-paclitaxel were approved as the first-line treatment of metastatic squamous NSCLC based on the results of KEYNOTE-407. On the strength of a series of successes in clinical trials, the approval of pembrolizumab plus chemotherapy covers more tumors, including gastroesophageal junction cancer (KEYNOTE-811), advanced triple-negative breast cancer (KEYNOTE-355), and esophageal cancer (KEYNOTE-590) (264–266). Meanwhile, anti-PD-L1-based chemoimmunotherapy such as atezolizumab plus chemotherapy and durvalumab combined with platinum plus etoposide treatment, has also received approval from the FDA in different tumors (170, 267). There is currently a rapidly growing number of clinical trials assessing chemoimmunotherapeutic regimens with the PD-1/PD-L1 inhibitor in clinical development but have not yet been approved by the FDA (166). The dose and sequence of administration require further evaluation to maximize the benefits of immunogenic chemotherapy.

### Combining anti− programmed cell death protein 1 (PD-1)/programmed cell death ligand 1 with radiotherapy

Based on the above-mentioned preclinical data suggesting the potential synergistic effect of combining radiotherapy with anti−PD−1/PD−L1, a mounting number of translations into clinical trials are ongoing, most of which are still in phase I or II. In addition, the majority of radioimmunotherapy regimens are based on stereotactic body radiotherapy (SBRT). For instance, in PEMBRO-RT, a multicenter randomized phase 2 study of 92 patients with advanced NSCLC, patients who received SBRT (three doses of 8 Gy) before pembrolizumab showed improved trends in OS, progression-free survival (PFS), and objective response rate (ORR) compared with the non-irradiated group (268). However, in a single-center, randomized, phase II trial (NCT02684253) for patients with metastatic or recurrent HNSCC, nivolumab plus SBRT showed no improvement in response compared with nivolumab single arm (269). Further research is needed to explore the best radioimmunotherapy options, including the dose, volume, fractionation, and sequence.

### Dual immune checkpoint blockade

The combination of ipilimumab (anti-CTLA-4) and nivolumab (anti-PD-1) is the first FDA-approved dual ICB treatment based on the results of CheckMate-067, CheckMate-069, and CheckMate-142 (151, 270). This combination is currently applied for the treatment of melanoma, RCC, HCC, PD-L1-positive NSCLC, MSI-H/dMMR colorectal cancer, and malignant pleural mesothelioma (3). Moreover, the FDA recently approved the first fixed-dose combination of nivolumab (Opdivo) and relatlimab (LAG-3 inhibitor) for unresectable or metastatic melanoma patients based on an appealing result from the phase-II/III RELATIVITY-047 trial. This trial demonstrated that the relatlimab–nivolumab combination yielded a progression-free survival rate of 10.1 months compared with 4.6 months in nivolumab monotherapy without new safety problems (271). Combinations of PD-1/PD-L1 blockers with other ICB are still in clinical trials. For instance, another CTLA-4 targeted monoclonal antibody, tremelimumab plus durvalumab, has entered phase 3 clinical trials in various malignancies, including small-cell lung cancer, high-risk urothelial carcinoma, advanced colorectal cancer, and advanced gastric and gastroesophageal junction adenocarcinoma, some of which received unsatisfactory results. No additional benefit was shown in combination (272–275). The severity and incidence of immune-related adverse events (irAEs), including colitis, thyroiditis, pneumonitis, and hypophysitis, have also been reported in the coblockade of PD-1/PD-L1 and CTLA-4 patients (276). In the primary analysis of the phase 2 CITYSCAPE trial, the TIGIT inhibitor tiragolumab plus atezolizumab (anti-PD-L1) showed improvement in PFS (stratified HR, 0.58; 95% CI, 0.38–0.89) in PD-L1-positive NSCLC patients (277).

### Combining immune checkpoint blockade with targeted therapies in cancer treatment

Preclinical and clinical studies have verified the synergetic effect of the angiogenesis inhibitor with anti−PD−1/PD−L1. Based on studies 309/KEYNOTE-775 (NCT03517449) and KEYNOTE581 (NCT02811861), lenvatinib plus pembrolizumab has been approved by the FDA in the treatment of advanced endometrial carcinoma and advanced RCC (278). The KEYNOTE-426 study revealed that patients receiving pembrolizumab plus axitinib gained statistically significant PFS, OS, and ORR improvement compared with sunitinib monotherapy, which promoted the approval of pembrolizumab plus axitinib as the first-line therapy for advanced RCC (279). In 2018, based on the IMpower150 trial (NCT02366143), atezolizumab with chemotherapy and bevacizumab was approved for the first-line treatment of metastatic non-squamous NSCLC (280). Additionally, atezolizumab combined with bevacizumab was approved in 2020 for unresectable hepatocellular carcinoma on the basis of the IMbrave150 trial (NCT03434379) (281). Moreover, the FDA approved axitinib plus avelumab (based on JAVELIN Renal 101) and cabozantinib plus nivolumab (based on CheckMate-9ER) for RCC initial-line treatment as well (282, 283).

Noteworthily, plenty of clinical trials are exploring the combination strategies of angiogenesis inhibitors and anti-PD-1/PD-L1 at present. The preliminary data of some combinations demonstrated favorable therapeutic effects such as camrelizumab plus apatinib in advanced triple-negative breast cancer (NCT03394287), advanced cervical cancer (NCT03816553), and advanced HCC (NCT03463876) and sintilimab plus anlotinib in advanced NSCLC (NCT03628521) and PD-L1-positive recurrent or metastatic cervical cancer (284). Subsequent phase 3 trials are necessary to confirm the effectiveness of these combination regimens.

Apart from angiogenesis inhibitors, various targeted therapies combined with anti-PD-1/PD-L1 are undergoing clinical trials, such as nivolumab plus erlotinib (EGFR) in NSCLC patients (NCT01454102), tislelizumab plus pamiparib (PARP) in solid tumor patients (NCT02660034), cobimetinib (MEK) plus atezolizumab in colorectal cancer patients (NCT02788279), nivolumab plus copanlisib (PI3K) in lymphoma and solid tumor patients (NCT03502733), and pembrolizumab plus abemaciclib (CDK4/6) in NSCLC and breast cancer patients (NCT02779751). Altogether, most clinical trials are still in phase I or II. Further research is needed to explore the efficacy of anti-PD-1/PD-L1-based combined strategies in phase 3 trials.

### Concluding remarks

ICB has revolutionized the field of cancer treatment. However, the initial wave of success on ICB is challenged by primary and acquired resistance. The number of patients benefiting from ICB is limited. Thus, a more detailed map of resistant mechanisms is reasonably necessary to develop coping strategies to improve clinical outcomes. Firstly, in this context, we primarily focus on the changes in the biological functions of CD8^+^ T cells to elucidate the underlying resistance mechanisms of ICB therapies. Based on the mechanical studies of both tumoral and systemic changes in the immune system, dozens of combinational regimens have been proposed, some of which exhibit potent antitumor activities in preclinical and clinical studies. Secondly, chemotherapy, VEGF/VEGFR-targeted therapy, and CTLA4-targeted treatment have been shown to be the most promising combinational options with anti-PD-1/PD-L1 therapy. They have great potential to improve the efficacy of ICB treatment in the condition of drug resistance. Nevertheless, only a tiny number of combinational strategies have been approved by the FDA, including anti-PD-1/PD-L1 plus chemotherapy, angiogenesis inhibitor, anti-CTLA-4, and anti-LAG-3 (**Table 2**). Overall, with a more profound elucidation of ICB resistance mechanisms, more novel clues of combinational strategies will emerge. Additional effort is needed to overcome barriers, including the occurrence of irAEs, the assessment of predictive biomarkers, and the definition of administration regimens such as dosage, timing, and sequence.

**Table 2:** Approved combination strategies with the PD-1/PDL1 inhibitor.

Combined strategy	anti−PD−1/PD−L1	Cancer type	Clinical trial	Approval time
Chemotherapy	Pembrolizumab	Metastatic non-squamous NSCLC	KEYNOTE-189 (NCT02578680)	08/20/2018
		Metastatic squamous NSCLC	KEYNOTE-407 (NCT02775435)	10/30/2018
		Metastatic TNBC	KEYNOTE-355 (NCT02819518)	11/13/2020
		Esophageal or GEJ carcinoma	KEYNOTE-590 (NCT03189719)	03/22/2021
		Cervical cancer	KEYNOTE-826 (NCT03635567)	10/13/2021
	Nivolumab	Metastatic gastric cancer and esophageal adenocarcinoma	CHECKMATE-649 (NCT02872116)	04/16/2021
	Atezolizumab	PD-L1 positive unresectable locally advanced or metastatic TNBC	IMpassion130 (NCT02425891)	03/18/2019
		ES-SCLC	IMpower133 (NCT02763579)	03/18/2019
		Metastatic NSCLC without EGFR/ALK aberrations	IMpower130 (NCT02367781)	12/03/2019
	Durvalumab	ES-SCLC	NCT03043872	03/27/2020
Axitinib	Pembrolizumab	Advanced RCC	KEYNOTE−426 (NCT02853331)	04/19/2019
	Avelumab	RCC	JAVELIN Renal 101 (NCT02684006)	05/14/2019
Lenvatinib	Pembrolizumab	Advanced endometrial carcinoma	KEYNOTE-775 (NCT03517449)	06/21/2021
Bevacizumab	Atezolizumab	Unresectable HCC	IMbrave150 (NCT03434379)	05/29/2020
Cabozantinib	Nivolumab	Advanced RCC	CHECKMATE-9ER (NCT03141177)	01/22/2021
Ipilimumab (anti-CTLA-4)	Nivolumab (anti-PD-1)	Metastatic melanoma	CheckMate-069	10/01/2015
		Intermediate- or poor-risk advanced RCC	CheckMate 214 (NCT02231749)	04/16/2018
		MSI-H/dMMR colorectal cancer	CHECKMATE 142 (NCT02060188)	07/10/2018
		HCC	CHECKMATE-040, (NCT01658878)	03/10/2020
		PD-L1-positive NSCLC	CHECKMATE-227 (NCT02477826)	05/15/2020
		Malignant pleural mesothelioma	CHECKMATE-743 (NCT02899299)	10/02/2020
Relatlimab (LAG-3 inhibitor)	Nivolumab	Unresectable or metastatic melanoma	RELATIVITY-047 (NCT03470922)	03/18/2022

NSCLC, non-small cell lung cancer; TNBC, triple-negative breast cancer; GEJ, gastroesophageal; ES-SCLC, extensive-stage small cell lung cancer; RCC, renal cell carcinoma; HCC, hepatocellular carcinoma.

## Author contributions

XTZ and YN jointly contributed to the first draft of the article, tables, and figures. XL provided assistance in making figures. YL, BA, and XH revised the manuscript. XZ conceived the presented idea, revised the manuscript again, and approved the final version. All authors approved this manuscript for publication.

## Funding

This work was supported by the National Natural Science Foundation of China (No. 81902662), the National Natural Science Foundation of China (No. 81821002), Sichuan Science and Technology Program 2021YJ0011, and Sichuan Science and Technology Program 2018YJ0609.

## Conflict of interest

The authors declare that the research was conducted in the absence of any commercial or financial relationships that could be construed as a potential conflict of interest.

## Publisher’s note

All claims expressed in this article are solely those of the authors and do not necessarily represent those of their affiliated organizations, or those of the publisher, the editors and the reviewers. Any product that may be evaluated in this article, or claim that may be made by its manufacturer, is not guaranteed or endorsed by the publisher.

## References

[1] Postow MA Callahan MK Wolchok JD. Immune checkpoint blockade in cancer therapy. J Clin Oncol (2015) 33(17):1974–82. doi: 10.1200/JCO.2014.59.4358 PMC4980573 25605845

[2] Sharma P Hu-Lieskovan S Wargo JA Ribas A. Primary, adaptive, and acquired resistance to cancer immunotherapy. Cell (2017) 168(4):707–23. doi: 10.1016/j.cell.2017.01.017 PMC5391692 28187290

[3] Vaddepally RK Kharel P Pandey R Garje R Chandra AB. Review of indications of fda-approved immune checkpoint inhibitors per nccn guidelines with the level of evidence. Cancers (Basel) (2020) 12(3):738. doi: 10.3390/cancers12030738 PMC7140028 32245016

[4] Schoenfeld AJ Hellmann MD. Acquired resistance to immune checkpoint inhibitors. Cancer Cell (2020) 37(4):443–55. doi: 10.1016/j.ccell.2020.03.017 PMC7182070 32289269

[5] Morad G Helmink BA Sharma P Wargo JA. Hallmarks of response, resistance, and toxicity to immune checkpoint blockade. Cell (2021) 184(21):5309–37. doi: 10.1016/j.cell.2021.09.020 PMC8767569 34624224

[6] Aldea M Andre F Marabelle A Dogan S Barlesi F Soria J-C. Overcoming resistance to tumor-targeted and immune-targeted therapies. Cancer Discovery (2021) 11(4):874–99. doi: 10.1158/2159-8290.CD-20-1638 33811122

[7] Bagchi S Yuan R Engleman EG. Immune checkpoint inhibitors for the treatment of cancer: Clinical impact and mechanisms of response and resistance. Annu Rev Pathol (2021) 16:223–49. doi: 10.1146/annurev-pathol-042020-042741 33197221

[8] Li J Stanger BZ. How tumor cell dedifferentiation drives immune evasion and resistance to immunotherapy. Cancer Res (2020) 80(19):4037–41. doi: 10.1158/0008-5472.CAN-20-1420 PMC7541560 32554552

[9] Arozarena I Wellbrock C. Phenotype plasticity as enabler of melanoma progression and therapy resistance. Nat Rev Cancer (2019) 19(7):377–91. doi: 10.1038/s41568-019-0154-4 31209265

[10] Perez-Guijarro E Yang HH Araya RE El Meskini R Michael HT Vodnala SK. Multimodel preclinical platform predicts clinical response of melanoma to immunotherapy. Nat Med (2020) 26(5):781–91. doi: 10.1038/s41591-020-0818-3 PMC8482620 32284588

[11] Horn LA Fousek K Palena C. Tumor plasticity and resistance to immunotherapy. Trends Cancer (2020) 6(5):432–41. doi: 10.1016/j.trecan.2020.02.001 PMC7192950 32348738

[12] Kalbasi A Ribas A. Tumour-intrinsic resistance to immune checkpoint blockade. Nat Rev Immunol (2020) 20(1):25–39. doi: 10.1038/s41577-019-0218-4 31570880 PMC8499690

[13] Horvath L Thienpont B Zhao L Wolf D Pircher A. Overcoming immunotherapy resistance in non-small cell lung cancer (Nsclc) - novel approaches and future outlook. Mol Cancer (2020) 19(1):141. doi: 10.1186/s12943-020-01260-z 32917214 PMC7488475

[14] Galluzzi L Humeau J Buque A Zitvogel L Kroemer G. Immunostimulation with chemotherapy in the era of immune checkpoint inhibitors. Nat Rev Clin Oncol (2020) 17(12):725–41. doi: 10.1038/s41571-020-0413-z 32760014

[15] Schaue D McBride WH. Opportunities and challenges of radiotherapy for treating cancer. Nat Rev Clin Oncol (2015) 12(9):527–40. doi: 10.1038/nrclinonc.2015.120 PMC8396062 26122185

[16] Morandi F Airoldi I. Hla-G and other immune checkpoint molecules as targets for novel combined immunotherapies. Int J Mol Sci (2022) 23(6):2925. doi: 10.3390/ijms23062925 35328349 PMC8948858

[17] Franco F Jaccard A Romero P Yu YR Ho PC. Metabolic and epigenetic regulation of T-cell exhaustion. Nat Metab (2020) 2(10):1001–12. doi: 10.1038/s42255-020-00280-9 32958939

[18] Routy B Le Chatelier E Derosa L Duong CPM Alou MT Daillere R. Gut microbiome influences efficacy of pd-1-Based immunotherapy against epithelial tumors. Science (2018) 359(6371):91–7. doi: 10.1126/science.aan3706 29097494

[19] Yi M Jiao D Qin S Chu Q Wu K Li A. Synergistic effect of immune checkpoint blockade and anti-angiogenesis in cancer treatment. Mol Cancer (2019) 18(1):60. doi: 10.1186/s12943-019-0974-6 30925919 PMC6441150

[20] Melief CJM. Mutation-specific T cells for immunotherapy of gliomas. N Engl J Med (2015) 372(20):1956–8. doi: 10.1056/NEJMcibr1501818 25970054

[21] Huang Y Shah S Qiao L. Tumor resistance to Cd8+ T cell-based therapeutic vaccination. Arch Immunol Ther Exp (Warsz) (2007) 55(4):205–17. doi: 10.1007/s00005-007-0029-3 17659376

[22] Lanzavecchia A Sallusto F. Progressive differentiation and selection of the fittest in the immune response. Nat Rev Immunol (2002) 2(12):982–7. doi: 10.1038/nri959 12461571

[23] Sade-Feldman M Yizhak K Bjorgaard SL Ray JP de Boer CG Jenkins RW. Defining T cell states associated with response to checkpoint immunotherapy in melanoma. Cell (2018) 175(4):998–1013.e20. doi: 10.1016/j.cell.2018.10.038 PMC6641984 30388456

[24] Li W Sun T Li M He Y Li L Wang L. Gnifdb: A neoantigen intrinsic feature database for glioma. Database (Oxford) (2022) 2022:baac004. doi: 10.1093/database/baac004 35150127 PMC9216533

[25] Strickler JH Hanks BA Khasraw M. Tumor mutational burden as a predictor of immunotherapy response: Is more always better? Clin Cancer Res (2021) 27(5):1236–41. doi: 10.1158/1078-0432.CCR-20-3054 PMC9912042 33199494

[26] Yarchoan M Hopkins A Jaffee EM. Tumor mutational burden and response rate to pd-1 inhibition. N Engl J Med (2017) 377(25):2500–1. doi: 10.1056/NEJMc1713444 PMC6549688 29262275

[27] Larkin J Chiarion-Sileni V Gonzalez R Grob JJ Cowey CL Lao CD. Combined nivolumab and ipilimumab or monotherapy in untreated melanoma. N Engl J Med (2015) 373(1):23–34. doi: 10.1056/NEJMoa1504030 26027431 PMC5698905

[28] Marabelle A Fakih M Lopez J Shah M Shapira-Frommer R Nakagawa K. Association of tumour mutational burden with outcomes in patients with advanced solid tumours treated with pembrolizumab: Prospective biomarker analysis of the multicohort, open-label, phase 2 keynote-158 study. Lancet Oncol (2020) 21(10):1353–65. doi: 10.1016/S1470-2045(20)30445-9 32919526

[29] von Loga K Woolston A Punta M Barber LJ Griffiths B Semiannikova M. Extreme intratumour heterogeneity and driver evolution in mismatch repair deficient gastro-oesophageal cancer. Nat Commun (2020) 11(1):139. doi: 10.1038/s41467-019-13915-7 31949146 PMC6965135

[30] Strickler JH Hanks BA Khasraw M. Tumor mutational burden as a predictor of immunotherapy response: Is more always better? Clin Cancer Res (2021) 27(5):1236–41. doi: 10.1158/1078-0432.CCR-20-3054 PMC9912042 33199494

[31] Henon C Blay JY Massard C Mir O Bahleda R Dumont S. Long lasting major response to pembrolizumab in a thoracic malignant rhabdoid-like Smarca4-deficient tumor. Ann Oncol (2019) 30(8):1401–3. doi: 10.1093/annonc/mdz160 31114851

[32] Gejman RS Chang AY Jones HF DiKun K Hakimi AA Schietinger A. Rejection of immunogenic tumor clones is limited by clonal fraction. Elife (2018) 7:e41090. doi: 10.7554/eLife.41090 30499773 PMC6269121

[33] Andor N Graham TA Jansen M Xia LC Aktipis CA Petritsch C. Pan-cancer analysis of the extent and consequences of intratumor heterogeneity. Nat Med (2016) 22(1):105–13. doi: 10.1038/nm.3984 PMC4830693 26618723

[34] Wolf Y Bartok O Patkar S Eli GB Cohen S Litchfield K. Uvb-induced tumor heterogeneity diminishes immune response in melanoma. Cell (2019) 179(1):219–235.e21. doi: 10.1016/j.cell.2019.08.032 PMC6863386 31522890

[35] McGranahan N Furness AJ Rosenthal R Ramskov S Lyngaa R Saini SK. Clonal neoantigens elicit T cell immunoreactivity and sensitivity to immune checkpoint blockade. Science (2016) 351(6280):1463–9. doi: 10.1126/science.aaf1490 PMC4984254 26940869

[36] Anagnostou V Smith KN Forde PM Niknafs N Bhattacharya R White J. Evolution of neoantigen landscape during immune checkpoint blockade in non-small cell lung cancer. Cancer Discovery (2017) 7(3):264–76. doi: 10.1158/2159-8290.CD-16-0828 PMC5733805 28031159

[37] Jamal-Hanjani M Wilson GA McGranahan N Birkbak NJ Watkins TBK Veeriah S. Tracking the evolution of non-Small-Cell lung cancer. N Engl J Med (2017) 376(22):2109–21. doi: 10.1056/NEJMoa1616288 28445112

[38] Blum JS Wearsch PA Cresswell P. Pathways of antigen processing. Annu Rev Immunol (2013) 31:443–73. doi: 10.1146/annurev-immunol-032712-095910 PMC4026165 23298205

[39] Hulpke S Tampé R. The mhc I loading complex: A multitasking machinery in adaptive immunity. Trends Biochem Sci (2013) 38(8):412–20. doi: 10.1016/j.tibs.2013.06.003 23849087

[40] Zaretsky JM Garcia-Diaz A Shin DS Escuin-Ordinas H Hugo W Hu-Lieskovan S. Mutations associated with acquired resistance to pd-1 blockade in melanoma. N Engl J Med (2016) 375(9):819–29. doi: 10.1056/NEJMoa1604958 PMC5007206 27433843

[41] Sade-Feldman M Jiao YJ Chen JH Rooney MS Barzily-Rokni M Eliane J-P. Resistance to checkpoint blockade therapy through inactivation of antigen presentation. Nat Commun (2017) 8(1):1136. doi: 10.1038/s41467-017-01062-w 29070816 PMC5656607

[42] Gettinger S Choi J Hastings K Truini A Datar I Sowell R. Impaired hla class I antigen processing and presentation as a mechanism of acquired resistance to immune checkpoint inhibitors in lung cancer. Cancer Discovery (2017) 7(12):1420–35. doi: 10.1158/2159-8290.CD-17-0593 PMC5718941 29025772

[43] Busch E Ahadova A Kosmalla K Bohaumilitzky L Pfuderer PL Ballhausen A. Mutations are linked to a distinct metastatic pattern and a favorable outcome in microsatellite-unstable stage iv gastrointestinal cancers. Front Oncol (2021) 11:669774. doi: 10.3389/fonc.2021.669774 34168989 PMC8219238

[44] Gurjao C Liu D Hofree M AlDubayan SH Wakiro I Su M-J. Intrinsic resistance to immune checkpoint blockade in a mismatch repair-deficient colorectal cancer. Cancer Immunol Res (2019) 7(8):1230–6. doi: 10.1158/2326-6066.CIR-18-0683 PMC6679789 31217164

[45] Kalbasi A Ribas A. Antigen presentation keeps trending in immunotherapy resistance. Clin Cancer Res (2018) 24(14):3239–41. doi: 10.1158/1078-0432.CCR-18-0698 PMC6050090 29674509

[46] Sivapalan L Anagnostou V. Genetic variation in antigen presentation and cancer immunotherapy. Immunity (2022) 55(1):3–6. doi: 10.1016/j.immuni.2021.12.010 35021056 PMC9844532

[47] Tran E Robbins PF Lu Y-C Prickett TD Gartner JJ Jia L. T-Cell transfer therapy targeting mutant kras in cancer. N Engl J Med (2016) 375(23):2255–62. doi: 10.1056/NEJMoa1609279 PMC5178827 27959684

[48] Saveanu L Carroll O Hassainya Y van Endert P. Complexity, contradictions, and conundrums: Studying post-proteasomal proteolysis in hla class I antigen presentation. Immunol Rev (2005) 207:42–59. doi: 10.1111/j.0105-2896.2005.00313.x 16181326

[49] Cabrera CM Jiménez P Cabrera T Esparza C Ruiz-Cabello F Garrido F. Total loss of mhc class I in colorectal tumors can be explained by two molecular pathways: Beta2-microglobulin inactivation in msi-positive tumors and Lmp7/Tap2 downregulation in msi-negative tumors. Tissue Antigens (2003) 61(3):211–9. doi: 10.1034/j.1399-0039.2003.00020.x 12694570

[50] Vitale M Rezzani R Rodella L Zauli G Grigolato P Cadei M. Hla class I antigen and transporter associated with antigen processing (Tap1 and Tap2) down-regulation in high-grade primary breast carcinoma lesions. Cancer Res (1998) 58(4):737–42. 9485029

[51] Zhang X Sabio E Krishna C Ma X Wang J Jiang H. Qa-1 modulates resistance to anti-Pd-1 immune checkpoint blockade in tumors with defects in antigen processing. Mol Cancer Res (2021) 19(6):1076–84. doi: 10.1158/1541-7786.MCR-20-0652 PMC8178214 33674442

[52] Vigneron N Ferrari V Van den Eynde BJ Cresswell P Leonhardt RM. Cytosolic processing governs tap-independent presentation of a critical melanoma antigen. J Immunol (2018) 201(7):1875–88. doi: 10.4049/jimmunol.1701479 PMC6457910 30135181

[53] Romero JM Jiménez P Cabrera T Cózar JM Pedrinaci S Tallada M. Coordinated downregulation of the antigen presentation machinery and hla class I/Beta2-microglobulin complex is responsible for hla-abc loss in bladder cancer. Int J Cancer (2005) 113(4):605–10. doi: 10.1002/ijc.20499 15455355

[54] López-Albaitero A Nayak JV Ogino T Machandia A Gooding W DeLeo AB. Role of antigen-processing machinery in the *in vitro* resistance of squamous cell carcinoma of the head and neck cells to recognition by ctl. J Immunol (2006) 176(6):3402–9. doi: 10.4049/jimmunol.176.6.3402 16517708

[55] Okada M Shimizu K Iyoda T Ueda S Shinga J Mochizuki Y. Pd-L1 expression affects neoantigen presentation. iScience (2020) 23(6):101238. doi: 10.1016/j.isci.2020.101238 32629606 PMC7322261

[56] Wangmo D Premsrirut PK Yuan C Morris WS Zhao X Subramanian S. Ackr4 in tumor cells regulates dendritic cell migration to tumor-draining lymph nodes and T-cell priming. Cancers (Basel) (2021) 13(19):5021. doi: 10.3390/cancers13195021 34638505 PMC8507805

[57] Liu X Shang X Li J Zhang S. The prognosis and immune checkpoint blockade efficacy prediction of tumor-infiltrating immune cells in lung cancer. Front Cell Dev Biol (2021) 9:707143. doi: 10.3389/fcell.2021.707143 34422829 PMC8370893

[58] Walker LSK Sansom DM. The emerging role of Ctla4 as a cell-extrinsic regulator of T cell responses. Nat Rev Immunol (2011) 11(12):852–63. doi: 10.1038/nri3108 22116087

[59] Maeda Y Nishikawa H Sugiyama D Ha D Hamaguchi M Saito T. Detection of self-reactive Cd8^+^T cells with an anergic phenotype in healthy individuals. Science (2014) 346(6216):1536–40. doi: 10.1126/science.aaa1292 25525252

[60] Narayanan S Vicent S Ponz-Sarvisé M. Pdac as an immune evasive disease: Can 3d model systems aid to tackle this clinical problem? Front Cell Dev Biol (2021) 9:787249. doi: 10.3389/fcell.2021.787249 34957115 PMC8703167

[61] Annels NE Simpson GR Denyer M Arif M Coffey M Melcher A. Oncolytic reovirus-mediated recruitment of early innate immune responses reverses immunotherapy resistance in prostate tumors. Mol Ther Oncolytics (2021) 20:434–46. doi: 10.1016/j.omto.2020.09.010 PMC7900644 33665363

[62] Salemizadeh Parizi M Salemizadeh Parizi F Abdolhosseini S Vanaei S Manzouri A Ebrahimzadeh F. Myeloid-derived suppressor cells (Mdscs) in brain cancer: Challenges and therapeutic strategies. Inflammopharmacology (2021) 29(6):1613–24. doi: 10.1007/s10787-021-00878-9 34613567

[63] Gajewski TF Woo S-R Zha Y Spaapen R Zheng Y Corrales L. Cancer immunotherapy strategies based on overcoming barriers within the tumor microenvironment. Curr Opin Immunol (2013) 25(2):268–76. doi: 10.1016/j.coi.2013.02.009 23579075

[64] Mühlberger M Janko C Unterweger H Friedrich RP Friedrich B Band J. Functionalization of T lymphocytes with citrate-coated superparamagnetic iron oxide nanoparticles for magnetically controlled immune therapy. Int J Nanomedicine (2019) 14:8421–32. doi: 10.2147/IJN.S218488 PMC6817714 31749616

[65] Chen DS Mellman I. Elements of cancer immunity and the cancer-immune set point. Nature (2017) 541(7637):321–30. doi: 10.1038/nature21349 28102259

[66] Peranzoni E Lemoine J Vimeux L Feuillet V Barrin S Kantari-Mimoun C. Macrophages impede Cd8 T cells from reaching tumor cells and limit the efficacy of anti-Pd-1 treatment. Proc Natl Acad Sci U.S.A. (2018) 115(17):E4041–E50. doi: 10.1073/pnas.1720948115 PMC5924916 29632196

[67] Peng W Chen JQ Liu C Malu S Creasy C Tetzlaff MT. Loss of pten promotes resistance to T cell-mediated immunotherapy. Cancer Discovery (2016) 6(2):202–16. doi: 10.1158/2159-8290.CD-15-0283 PMC4744499 26645196

[68] Karoulia Z Gavathiotis E Poulikakos PI. New perspectives for targeting raf kinase in human cancer. Nat Rev Cancer (2017) 17(11):676–91. doi: 10.1038/nrc.2017.79 PMC6000833 28984291

[69] Kimura ET Nikiforova MN Zhu Z Knauf JA Nikiforov YE Fagin JA. High prevalence of braf mutations in thyroid cancer: Genetic evidence for constitutive activation of the Ret/Ptc-Ras-Braf signaling pathway in papillary thyroid carcinoma. Cancer Res (2003) 63(7):1454–7. 12670889

[70] Rajagopalan H Bardelli A Lengauer C Kinzler KW Vogelstein B Velculescu VE. Tumorigenesis: Raf/Ras oncogenes and mismatch-repair status. Nature (2002) 418(6901):934. doi: 10.1038/418934a 12198537

[71] Wilmott JS Long GV Howle JR Haydu LE Sharma RN Thompson JF. Selective braf inhibitors induce marked T-cell infiltration into human metastatic melanoma. Clin Cancer Res (2012) 18(5):1386–94. doi: 10.1158/1078-0432.CCR-11-2479 22156613

[72] Skoulidis F Goldberg ME Greenawalt DM Hellmann MD Awad MM Gainor JF. Mutations and pd-1 inhibitor resistance in -mutant lung adenocarcinoma. Cancer Discovery (2018) 8(7):822–35. doi: 10.1158/2159-8290.CD-18-0099 PMC6030433 29773717

[73] Xu Y-P Lv L Liu Y Smith MD Li W-C Tan X-M. Tumor suppressor Tet2 promotes cancer immunity and immunotherapy efficacy. J Clin Invest (2019) 129(10):4316–31. doi: 10.1172/JCI129317 PMC6763236 31310587

[74] Dong Z-Y Zhang J-T Liu S-Y Su J Zhang C Xie Z. Egfr mutation correlates with uninflamed phenotype and weak immunogenicity, causing impaired response to pd-1 blockade in non-small cell lung cancer. Oncoimmunology (2017) 6(11):e1356145. doi: 10.1080/2162402X.2017.1356145 29147605 PMC5674946

[75] Huang H Langenkamp E Georganaki M Loskog A Fuchs PF Dieterich LC. Vegf suppresses T-lymphocyte infiltration in the tumor microenvironment through inhibition of nf-κb-Induced endothelial activation. FASEB J (2015) 29(1):227–38. doi: 10.1096/fj.14-250985 25361735

[76] Bruand M Barras D Mina M Ghisoni E Morotti M Lanitis E. Cell-autonomous inflammation of Brca1-deficient ovarian cancers drives both tumor-intrinsic immunoreactivity and immune resistance *Via* sting. Cell Rep (2021) 36(3):109412. doi: 10.1016/j.celrep.2021.109412 34289354 PMC8371260

[77] Li X He G Liu J Yan M Shen M Xu L. Ccl2-mediated monocytes regulate immune checkpoint blockade resistance in pancreatic cancer. Int Immunopharmacol. (2022) 106:108598. doi: 10.1016/j.intimp.2022.108598 35183036

[78] Tauriello DVF Palomo-Ponce S Stork D Berenguer-Llergo A Badia-Ramentol J Iglesias M. Tgfβ drives immune evasion in genetically reconstituted colon cancer metastasis. Nature (2018) 554(7693):538–43. doi: 10.1038/nature25492 29443964

[79] Mariathasan S Turley SJ Nickles D Castiglioni A Yuen K Wang Y. Tgfβ attenuates tumour response to pd-L1 blockade by contributing to exclusion of T cells. Nature (2018) 554(7693):544–8. doi: 10.1038/nature25501 PMC6028240 29443960

[80] Shaul ME Fridlender ZG. The dual role of neutrophils in cancer. Semin Immunol (2021) 57:101582. doi: 10.1016/j.smim.2021.101582 34974960

[81] Kim IS Gao Y Welte T Wang H Liu J Janghorban M. Immuno-subtyping of breast cancer reveals distinct myeloid cell profiles and immunotherapy resistance mechanisms. Nat Cell Biol (2019) 21(9):1113–26. doi: 10.1038/s41556-019-0373-7 PMC6726554 31451770

[82] Chen J Sun H-W Yang Y-Y Chen H-T Yu X-J Wu W-C. Reprogramming immunosuppressive myeloid cells by activated T cells promotes the response to anti-Pd-1 therapy in colorectal cancer. Signal Transduct. Target Ther (2021) 6(1):4. doi: 10.1038/s41392-020-00377-3 33414378 PMC7791142

[83] Meyer C Cagnon L Costa-Nunes CM Baumgaertner P Montandon N Leyvraz L. Frequencies of circulating mdsc correlate with clinical outcome of melanoma patients treated with ipilimumab. Cancer Immunol Immunother. (2014) 63(3):247–57. doi: 10.1007/s00262-013-1508-5 PMC11029062 24357148

[84] Movahedi K Guilliams M Van den Bossche J Van den Bergh R Gysemans C Beschin A. Identification of discrete tumor-induced myeloid-derived suppressor cell subpopulations with distinct T cell-suppressive activity. Blood (2008) 111(8):4233–44. doi: 10.1182/blood-2007-07-099226 18272812

[85] Liu C-Y Wang Y-M Wang C-L Feng P-H Ko H-W Liu Y-H. Population alterations of l-arginase- and inducible nitric oxide synthase-expressed Cd11b+/Cd14^-^/Cd15+/Cd33+ myeloid-derived suppressor cells and Cd8+ T lymphocytes in patients with advanced-stage non-small cell lung cancer. J Cancer Res Clin Oncol (2010) 136(1):35–45. doi: 10.1007/s00432-009-0634-0 19572148 PMC11827779

[86] Veglia F Tyurin VA Blasi M De Leo A Kossenkov AV Donthireddy L. Fatty acid transport protein 2 reprograms neutrophils in cancer. Nature (2019) 569(7754):73–8. doi: 10.1038/s41586-019-1118-2 PMC6557120 30996346

[87] Thorn M Guha P Cunetta M Espat NJ Miller G Junghans RP. Tumor-associated gm-csf overexpression induces immunoinhibitory molecules *Via* Stat3 in myeloid-suppressor cells infiltrating liver metastases. Cancer Gene Ther (2016) 23(6):188–98. doi: 10.1038/cgt.2016.19 27199222

[88] Adeshakin AO Liu W Adeshakin FO Afolabi LO Zhang M Zhang G. Regulation of ros in myeloid-derived suppressor cells through targeting fatty acid transport protein 2 enhanced anti-Pd-L1 tumor immunotherapy. Cell Immunol (2021) 362:104286. doi: 10.1016/j.cellimm.2021.104286 33524739

[89] DeNardo DG Ruffell B. Macrophages as regulators of tumour immunity and immunotherapy. Nat Rev Immunol (2019) 19(6):369–82. doi: 10.1038/s41577-019-0127-6 PMC7339861 30718830

[90] Orlikowsky T Dannecker GE Wang Z Horowitz H Niethammer D Hoffmann MK. Activation or destruction of T cells. Via Macrophages. Pathobiol. (1999) 67(5-6):298–301. doi: 10.1159/000028084 10725807

[91] Mantovani A Sozzani S Locati M Allavena P Sica A. Macrophage polarization: Tumor-associated macrophages as a paradigm for polarized M2 mononuclear phagocytes. Trends Immunol (2002) 23(11):549–55. doi: 10.1016/S1471-4906(02)02302-5 12401408

[92] Arlauckas SP Garris CS Kohler RH Kitaoka M Cuccarese MF Yang KS. *In vivo* imaging reveals a tumor-associated macrophage-mediated resistance pathway in anti-Pd-1 therapy. Sci Transl Med (2017) 9(389):eaal3604. doi: 10.1126/scitranslmed.aal3604 28490665 PMC5734617

[93] Ruffell B Chang-Strachan D Chan V Rosenbusch A Ho CMT Pryer N. Macrophage il-10 blocks Cd8+ T cell-dependent responses to chemotherapy by suppressing il-12 expression in intratumoral dendritic cells. Cancer Cell (2014) 26(5):623–37. doi: 10.1016/j.ccell.2014.09.006 PMC4254570 25446896

[94] Smith LK Boukhaled GM Condotta SA Mazouz S Guthmiller JJ Vijay R. Interleukin-10 directly inhibits Cd8 T cell function by enhancing n-glycan branching to decrease antigen sensitivity. Immunity (2018) 48(2):299–312.e5. doi: 10.1016/j.immuni.2018.01.006 PMC5935130 29396160

[95] Zerdes I Wallerius M Sifakis EG Wallmann T Betts S Bartish M. Stat3 activity promotes programmed-death ligand 1 expression and suppresses immune responses in breast cancer. Cancers (Basel) (2019) 11(10):1479. doi: 10.3390/cancers11101479 PMC6827034 31581535

[96] Singhal S Stadanlick J Annunziata MJ Rao AS Bhojnagarwala PS O'Brien S. Human tumor-associated Monocytes/Macrophages and their regulation of T cell responses in early-stage lung cancer. Sci Transl Med (2019) 11(479):eaat1500. doi: 10.1126/scitranslmed.aat1500 30760579 PMC6800123

[97] Darby IA Laverdet B Bonté F Desmoulière A. Fibroblasts and myofibroblasts in wound healing. Clin Cosmet Investig Dermatol (2014) 7:301–11. doi: 10.2147/CCID.S50046 PMC4226391 25395868

[98] Kuzet S-E Gaggioli C. Fibroblast activation in cancer: When seed fertilizes soil. Cell Tissue Res (2016) 365(3):607–19. doi: 10.1007/s00441-016-2467-x 27474009

[99] Gardner H Strehlow D Bradley L Widom R Farina A de Fougerolles A. Global expression analysis of the fibroblast transcriptional response to tgfbeta. Clin Exp Rheumatol (2004) 22(3 Suppl 33):S47–57. 15344598

[100] Khalili JS Liu S Rodríguez-Cruz TG Whittington M Wardell S Liu C. Oncogenic Braf(V600e) promotes stromal cell-mediated immunosuppression *Via* induction of interleukin-1 in melanoma. Clin Cancer Res (2012) 18(19):5329–40. doi: 10.1158/1078-0432.CCR-12-1632 PMC3463754 22850568

[101] Lakins MA Ghorani E Munir H Martins CP Shields JD. Cancer-associated fibroblasts induce antigen-specific deletion of Cd8 T cells to protect tumour cells. Nat Commun (2018) 9(1):948. doi: 10.1038/s41467-018-03347-0 29507342 PMC5838096

[102] Gorchs L Fernández Moro C Bankhead P Kern KP Sadeak I Meng Q. Human pancreatic carcinoma-associated fibroblasts promote expression of Co-inhibitory markers on Cd4 and Cd8 T-cells. Front Immunol (2019) 10:847. doi: 10.3389/fimmu.2019.00847 31068935 PMC6491453

[103] Cheng Y Li H Deng Y Tai Y Zeng K Zhang Y. Cancer-associated fibroblasts induce Pdl1+ neutrophils through the Il6-Stat3 pathway that foster immune suppression in hepatocellular carcinoma. Cell Death Dis (2018) 9(4):422. doi: 10.1038/s41419-018-0458-4 29556041 PMC5859264

[104] Li Z Zhou J Zhang J Li S Wang H Du J. Cancer-associated fibroblasts promote pd-L1 expression in mice cancer cells *Via* secreting Cxcl5. Int J Cancer (2019) 145(7):1946–57. doi: 10.1002/ijc.32278 PMC6767568 30873585

[105] Curiel TJ Coukos G Zou L Alvarez X Cheng P Mottram P. Specific recruitment of regulatory T cells in ovarian carcinoma fosters immune privilege and predicts reduced survival. Nat Med (2004) 10(9):942–9. doi: 10.1038/nm1093 15322536

[106] Ma GF Miao Q Liu YM Gao H Lian JJ Wang YN. High Foxp3 expression in tumour cells predicts better survival in gastric cancer and its role in tumour microenvironment. Br J Cancer (2014) 110(6):1552–60. doi: 10.1038/bjc.2014.47 PMC3960619 24548868

[107] Shimizu J Yamazaki S Takahashi T Ishida Y Sakaguchi S. Stimulation of Cd25(+)Cd4(+) regulatory T cells through gitr breaks immunological self-tolerance. Nat Immunol (2002) 3(2):135–42. doi: 10.1038/ni759 11812990

[108] Bennett CL Christie J Ramsdell F Brunkow ME Ferguson PJ Whitesell L. The immune dysregulation, polyendocrinopathy, enteropathy, X-linked syndrome (Ipex) is caused by mutations of Foxp3. Nat Genet (2001) 27(1):20–1. doi: 10.1038/83713 11137993

[109] Zhang Y Maksimovic J Huang B De Souza DP Naselli G Chen H. Cord blood Cd8 T cells have a natural propensity to express il-4 in a fatty acid metabolism and caspase activation-dependent manner. Front Immunol (2018) 9:879. doi: 10.3389/fimmu.2018.00879 29922282 PMC5996926

[110] Pereira LMS Gomes STM Ishak R Vallinoto ACR. Regulatory T cell and forkhead box protein 3 as modulators of immune homeostasis. Front Immunol (2017) 8:605. doi: 10.3389/fimmu.2017.00605 28603524 PMC5445144

[111] Ren Z Zhang A Sun Z Liang Y Ye J Qiao J. Selective delivery of low-affinity il-2 to pd-1+ T cells rejuvenates antitumor immunity with reduced toxicity. J Clin Invest (2022) 132(3):e153604. doi: 10.1172/JCI153604 35104810 PMC8803347

[112] Camisaschi C Casati C Rini F Perego M De Filippo A Triebel F. Lag-3 expression defines a subset of Cd4(+)Cd25(High)Foxp3(+) regulatory T cells that are expanded at tumor sites. J Immunol (2010) 184(11):6545–51. doi: 10.4049/jimmunol.0903879 20421648

[113] Linterman MA Pierson W Lee SK Kallies A Kawamoto S Rayner TF. Foxp3+ follicular regulatory T cells control the germinal center response. Nat Med (2011) 17(8):975–82. doi: 10.1038/nm.2425 PMC3182542 21785433

[114] Sage PT Francisco LM Carman CV Sharpe AH. The receptor pd-1 controls follicular regulatory T cells in the lymph nodes and blood. Nat Immunol (2013) 14(2):152–61. doi: 10.1038/ni.2496 PMC3788614 23242415

[115] Vanderleyden I Fra-Bido SC Innocentin S Stebegg M Okkenhaug H Evans-Bailey N. Follicular regulatory T cells can access the germinal center independently of Cxcr5. Cell Rep (2020) 30(3):611–619.e4. doi: 10.1016/j.celrep.2019.12.076 PMC6988108 31968240

[116] Eschweiler S Clarke J Ramírez-Suástegui C Panwar B Madrigal A Chee SJ. Intratumoral follicular regulatory T cells curtail anti-Pd-1 treatment efficacy. Nat Immunol (2021) 22(8):1052–63. doi: 10.1038/s41590-021-00958-6 PMC8434898 34168370

[117] Zappasodi R Budhu S Hellmann MD Postow MA Senbabaoglu Y Manne S. Non-conventional inhibitory Cd4foxp3pd-1 T cells as a biomarker of immune checkpoint blockade activity. Cancer Cell (2018) 33(6):1017–1032.e7. doi: 10.1016/j.ccell.2018.05.009 PMC6648657 29894689

[118] Wherry EJ. T Cell exhaustion. Nat Immunol (2011) 12(6):492–9. doi: 10.1038/ni.2035 21739672

[119] Konen JM Rodriguez BL Fradette JJ Gibson L Davis D Minelli R. Ntrk1 promotes resistance to pd-1 checkpoint blockade in mesenchymal Kras/P53 mutant lung cancer. Cancers (Basel) (2019) 11(4):462. doi: 10.3390/cancers11040462 PMC6521201 30986992

[120] Koyama S Akbay EA Li YY Herter-Sprie GS Buczkowski KA Richards WG. Adaptive resistance to therapeutic pd-1 blockade is associated with upregulation of alternative immune checkpoints. Nat Commun (2016) 7:10501. doi: 10.1038/ncomms10501 26883990 PMC4757784

[121] Shayan G Srivastava R Li J Schmitt N Kane LP Ferris RL. Adaptive resistance to anti-Pd1 therapy by Tim-3 upregulation is mediated by the Pi3k-akt pathway in head and neck cancer. Oncoimmunology (2017) 6(1):e1261779. doi: 10.1080/2162402X.2016.1261779 28197389 PMC5283618

[122] Huang R-Y Francois A McGray AR Miliotto A Odunsi K. Compensatory upregulation of pd-1, lag-3, and ctla-4 limits the efficacy of single-agent checkpoint blockade in metastatic ovarian cancer. Oncoimmunology (2017) 6(1):e1249561. doi: 10.1080/2162402X.2016.1249561 28197366 PMC5283642

[123] Thommen DS Schreiner J Müller P Herzig P Roller A Belousov A. Progression of lung cancer is associated with increased dysfunction of T cells defined by coexpression of multiple inhibitory receptors. Cancer Immunol Res (2015) 3(12):1344–55. doi: 10.1158/2326-6066.CIR-15-0097 26253731

[124] Kakavand H Jackett LA Menzies AM Gide TN Carlino MS Saw RPM. Negative immune checkpoint regulation by vista: A mechanism of acquired resistance to anti-Pd-1 therapy in metastatic melanoma patients. Mod Pathol (2017) 30(12):1666–76. doi: 10.1038/modpathol.2017.89 28776578

[125] Bauer C Kühnemuth B Duewell P Ormanns S Gress T Schnurr M. Prevailing over T cell exhaustion: New developments in the immunotherapy of pancreatic cancer. Cancer Lett (2016) 381(1):259–68. doi: 10.1016/j.canlet.2016.02.057 26968250

[126] Farhood B Najafi M Mortezaee K. Cd8 cytotoxic T lymphocytes in cancer immunotherapy: A review. J Cell Physiol (2019) 234(6):8509–21. doi: 10.1002/jcp.27782 30520029

[127] Kearney CJ Vervoort SJ Hogg SJ Ramsbottom KM Freeman AJ Lalaoui N. Tumor immune evasion arises through loss of tnf sensitivity. Sci Immunol (2018) 3(23):eaar3451. doi: 10.1126/sciimmunol.aar3451 29776993

[128] Dominiecki ME Beatty GL Pan Z-K Neeson P Paterson Y. Tumor sensitivity to ifn-gamma is required for successful antigen-specific immunotherapy of a transplantable mouse tumor model for hpv-transformed tumors. Cancer Immunol Immunother (2005) 54(5):477–88. doi: 10.1007/s00262-004-0610-0 PMC11032979 15750832

[129] Ikeda H Old LJ Schreiber RD. The roles of ifn gamma in protection against tumor development and cancer immunoediting. Cytokine Growth Factor Rev (2002) 13(2):95–109. doi: 10.1016/S1359-6101(01)00038-7 11900986

[130] Bach EA Aguet M Schreiber RD. The ifn gamma receptor: A paradigm for cytokine receptor signaling. Annu Rev Immunol (1997) 15:563–91. doi: 10.1146/annurev.immunol.15.1.563 9143700

[131] Gao J Shi LZ Zhao H Chen J Xiong L He Q. Loss of ifn-Γ pathway genes in tumor cells as a mechanism of resistance to anti-Ctla-4 therapy. Cell (2016) 167(2):397–404.e9. doi: 10.1016/j.cell.2016.08.069 PMC5088716 27667683

[132] Shin DS Zaretsky JM Escuin-Ordinas H Garcia-Diaz A Hu-Lieskovan S Kalbasi A. Primary resistance to pd-1 blockade mediated by Jak1/2 mutations. Cancer Discovery (2017) 7(2):188–201. doi: 10.1158/2159-8290.CD-16-1223 27903500 PMC5296316

[133] Vredevoogd DW Kuilman T Ligtenberg MA Boshuizen J Stecker KE de Bruijn B. Augmenting immunotherapy impact by lowering tumor tnf cytotoxicity threshold. Cell (2019) 178(3):585–599.e15. doi: 10.1016/j.cell.2019.06.014 31303383

[134] Herrera FG Bourhis J Coukos G. Radiotherapy combination opportunities leveraging immunity for the next oncology practice. CA Cancer J Clin (2017) 67(1):65–85. doi: 10.3322/caac.21358 27570942

[135] Woller N Gurlevik E Fleischmann-Mundt B Schumacher A Knocke S Kloos AM. Viral infection of tumors overcomes resistance to pd-1-Immunotherapy by broadening neoantigenome-directed T-cell responses. Mol Ther (2015) 23(10):1630–40. doi: 10.1038/mt.2015.115 PMC4817928 26112079

[136] Turner TB Meza-Perez S Londono A Katre A Peabody JE Smith HJ. Epigenetic modifiers upregulate mhc ii and impede ovarian cancer tumor growth. Oncotarget (2017) 8(27):44159–70. doi: 10.18632/oncotarget.17395 PMC5546470 28498806

[137] Mazzone R Zwergel C Mai A Valente S. Epi-drugs in combination with immunotherapy: A new avenue to improve anticancer efficacy. Clin Epigenet (2017) 9:59. doi: 10.1186/s13148-017-0358-y PMC5450222 28572863

[138] Lv M Chen M Zhang R Zhang W Wang C Zhang Y. Manganese is critical for antitumor immune responses *Via* cgas-sting and improves the efficacy of clinical immunotherapy. Cell Res (2020) 30(11):966–79. doi: 10.1038/s41422-020-00395-4 PMC7785004 32839553

[139] Kalbasi A Tariveranmoshabad M Hakimi K Kremer S Campbell KM Funes JM. Uncoupling interferon signaling and antigen presentation to overcome immunotherapy resistance due to Jak1 loss in melanoma. Sci Transl Med (2020) 12(565):eabb0152. doi: 10.1126/scitranslmed.abb0152 33055240 PMC8053376

[140] Ribas A Medina T Kummar S Amin A Kalbasi A Drabick JJ. Sd-101 in combination with pembrolizumab in advanced melanoma: Results of a phase ib, multicenter study. Cancer Discovery (2018) 8(10):1250–7. doi: 10.1158/2159-8290.CD-18-0280 PMC6719557 30154193

[141] Salmon H Idoyaga J Rahman A Leboeuf M Remark R Jordan S. Expansion and activation of Cd103(+) dendritic cell progenitors at the tumor site enhances tumor responses to therapeutic pd-L1 and braf inhibition. Immunity (2016) 44(4):924–38. doi: 10.1016/j.immuni.2016.03.012 PMC4980762 27096321

[142] Peng W Chen JQ Liu C Malu S Creasy C Tetzlaff MT. Loss of pten promotes resistance to T cell-mediated immunotherapy. Cancer Discovery (2016) 6(2):202–16. doi: 10.1158/2159-8290.CD-15-0283 PMC4744499 26645196

[143] Wall JA Meza-Perez S Scalise CB Katre A Londoño AI Turbitt WJ. Manipulating the Wnt/β-catenin signaling pathway to promote anti-tumor immune infiltration into the tme to sensitize ovarian cancer to icb therapy. Gynecol. Oncol (2021) 160(1):285–94. doi: 10.1016/j.ygyno.2020.10.031 PMC9107782 33168307

[144] Ribas A Lawrence D Atkinson V Agarwal S Miller WH Jr. Carlino MS. Combined braf and mek inhibition with pd-1 blockade immunotherapy in braf-mutant melanoma. Nat Med (2019) 25(6):936–40. doi: 10.1038/s41591-019-0476-5 PMC8562134 31171879

[145] Goel S DeCristo MJ Watt AC BrinJones H Sceneay J Li BB. Cdk4/6 inhibition triggers anti-tumour immunity. Nature (2017) 548(7668):471–5. doi: 10.1038/nature23465 PMC5570667 28813415

[146] Mariathasan S Turley SJ Nickles D Castiglioni A Yuen K Wang Y. Tgfbeta attenuates tumour response to pd-L1 blockade by contributing to exclusion of T cells. Nature (2018) 554(7693):544–8. doi: 10.1038/nature25501 PMC6028240 29443960

[147] Schmittnaegel M Rigamonti N Kadioglu E Cassara A Wyser Rmili C Kiialainen A. Dual angiopoietin-2 and vegfa inhibition elicits antitumor immunity that is enhanced by pd-1 checkpoint blockade. Sci Transl Med (2017) 9(385):eaak9670. doi: 10.1126/scitranslmed.aak9670 28404865

[148] Klug F Prakash H Huber PE Seibel T Bender N Halama N. Low-dose irradiation programs macrophage differentiation to an Inos(+)/M1 phenotype that orchestrates effective T cell immunotherapy. Cancer Cell (2013) 24(5):589–602. doi: 10.1016/j.ccr.2013.09.014 24209604

[149] Sun M Gu P Yang Y Yu L Jiang Z Li J. Mesoporous silica nanoparticles inflame tumors to overcome anti-Pd-1 resistance through Tlr4-nfkappab axis. J Immunother Cancer (2021) 9(6):e002508. doi: 10.1136/jitc-2021-002508 34117115 PMC8202116

[150] Grosser R Cherkassky L Chintala N Adusumilli PS. Combination immunotherapy with car T cells and checkpoint blockade for the treatment of solid tumors. Cancer Cell (2019) 36(5):471–82. doi: 10.1016/j.ccell.2019.09.006 PMC7171534 31715131

[151] Rotte A. Combination of ctla-4 and pd-1 blockers for treatment of cancer. J Exp Clin Cancer Res (2019) 38(1):255. doi: 10.1186/s13046-019-1259-z 31196207 PMC6567914

[152] Mayes PA Hance KW Hoos A. The promise and challenges of immune agonist antibody development in cancer. Nat Rev Drug Discovery (2018) 17(7):509–27. doi: 10.1038/nrd.2018.75 29904196

[153] Han HS Jeong S Kim H Kim HD Kim AR Kwon M. Tox-expressing terminally exhausted tumor-infiltrating Cd8(+) T cells are reinvigorated by Co-blockade of pd-1 and tigit in bladder cancer. Cancer Lett (2021) 499:137–47. doi: 10.1016/j.canlet.2020.11.035 33249194

[154] Ghoneim HE Fan Y Moustaki A Abdelsamed HA Dash P Dogra P. *De novo* epigenetic programs inhibit pd-1 blockade-mediated T cell rejuvenation. Cell (2017) 170(1):142–57.e19. doi: 10.1016/j.cell.2017.06.007 28648661 PMC5568784

[155] Buck MD O'Sullivan D Klein Geltink RI Curtis JD Chang CH Sanin DE. Mitochondrial dynamics controls T cell fate through metabolic programming. Cell (2016) 166(1):63–76. doi: 10.1016/j.cell.2016.05.035 27293185 PMC4974356

[156] Ravi R Noonan KA Pham V Bedi R Zhavoronkov A Ozerov IV. Bifunctional immune checkpoint-targeted antibody-ligand traps that simultaneously disable tgfbeta enhance the efficacy of cancer immunotherapy. Nat Commun (2018) 9(1):741. doi: 10.1038/s41467-017-02696-6 29467463 PMC5821872

[157] Neubert NJ Schmittnaegel M Bordry N Nassiri S Wald N Martignier C. T Cell-induced Csf1 promotes melanoma resistance to Pd1 blockade. Sci Transl Med (2018) 10(436):eaan3311. doi: 10.1126/scitranslmed.aan3311 29643229 PMC5957531

[158] Zhou Q Liang J Yang T Liu J Li B Li Y. Carfilzomib modulates tumor microenvironment to potentiate immune checkpoint therapy for cancer. EMBO Mol Med (2022) 14(1):e14502. doi: 10.15252/emmm.202114502 34898004 PMC8749493

[159] Cheng Y Li H Deng Y Tai Y Zeng K Zhang Y. Cancer-associated fibroblasts induce Pdl1+ neutrophils through the Il6-Stat3 pathway that foster immune suppression in hepatocellular carcinoma. Cell Death Dis (2018) 9(4):422. doi: 10.1038/s41419-018-0458-4 29556041 PMC5859264

[160] Ji D Song C Li Y Xia J Wu Y Jia J. Combination of radiotherapy and suppression of tregs enhances abscopal antitumor effect and inhibits metastasis in rectal cancer. J Immunother Cancer (2020) 8(2):e000826. doi: 10.1136/jitc-2020-000826 33106387 PMC7592256

[161] Derosa L Routy B Desilets A Daillère R Terrisse S Kroemer G. Microbiota-centered interventions: The next breakthrough in immuno-oncology? Cancer Discovery (2021) 11(10):2396–412. doi: 10.1158/2159-8290.Cd-21-0236 34400407

[162] Mole RH. Whole body irradiation; radiobiology or medicine? Br J Radiol (1953) 26(305):234–41. doi: 10.1259/0007-1285-26-305-234 13042090

[163] Ngwa W Irabor OC Schoenfeld JD Hesser J Demaria S Formenti SC. Using immunotherapy to boost the abscopal effect. Nat Rev Cancer (2018) 18(5):313–22. doi: 10.1038/nrc.2018.6 PMC5912991 29449659

[164] Takahashi J Nagasawa S. Immunostimulatory effects of radiotherapy for local and systemic control of melanoma: A review. Int J Mol Sci (2020) 21(23):9324. doi: 10.3390/ijms21239324 PMC7730562 33297519

[165] Sharma A Bode B Wenger RH Lehmann K Sartori AA Moch H. Gamma-radiation promotes immunological recognition of cancer cells through increased expression of cancer-testis antigens *in vitro* and *in vivo* . PloS One (2011) 6(11):e28217. doi: 10.1371/journal.pone.0028217 22140550 PMC3226680

[166] Salas-Benito D Perez-Gracia JL Ponz-Sarvise M Rodriguez-Ruiz ME Martinez-Forero I Castanon E. Paradigms on immunotherapy combinations with chemotherapy. Cancer Discovery (2021) 11(6):1353–67. doi: 10.1158/2159-8290.CD-20-1312 33712487

[167] Grimaldi A Cammarata I Martire C Focaccetti C Piconese S Buccilli M. Combination of chemotherapy and pd-1 blockade induces T cell responses to tumor non-mutated neoantigens. Commun Biol (2020) 3(1):85. doi: 10.1038/s42003-020-0811-x 32099064 PMC7042341

[168] Ma X Yang S Zhang T Wang S Yang Q Xiao Y. Bioresponsive immune-Booster-Based prodrug nanogel for cancer immunotherapy. Acta Pharm Sin B (2022) 12(1):451–66. doi: 10.1016/j.apsb.2021.05.016 PMC8800001 35127398

[169] Mathew M Enzler T Shu CA Rizvi NA. Combining chemotherapy with pd-1 blockade in nsclc. Pharmacol Ther (2018) 186:130–7. doi: 10.1016/j.pharmthera.2018.01.003 29352857

[170] Schmid P Rugo HS Adams S Schneeweiss A Barrios CH Iwata H. Atezolizumab plus nab-paclitaxel as first-line treatment for unresectable, locally advanced or metastatic triple-negative breast cancer (Impassion130): Updated efficacy results from a randomised, double-blind, placebo-controlled, phase 3 trial. Lancet Oncol (2020) 21(1):44–59. doi: 10.1016/S1470-2045(19)30689-8 31786121

[171] Russell L Peng KW Russell SJ Diaz RM. Oncolytic viruses: Priming time for cancer immunotherapy. BioDrugs (2019) 33(5):485–501. doi: 10.1007/s40259-019-00367-0 31321623 PMC6790338

[172] Bommareddy PK Shettigar M Kaufman HL. Integrating oncolytic viruses in combination cancer immunotherapy. Nat Rev Immunol (2018) 18(8):498–513. doi: 10.1038/s41577-018-0014-6 29743717

[173] Twumasi-Boateng K Pettigrew JL Kwok YYE Bell JC Nelson BH. Oncolytic viruses as engineering platforms for combination immunotherapy. Nat Rev Cancer (2018) 18(7):419–32. doi: 10.1038/s41568-018-0009-4 29695749

[174] Takaki H Cornelis F Kako Y Kobayashi K Kamikonya N Yamakado K. Thermal ablation and immunomodulation: From preclinical experiments to clinical trials. Diagn Interv Imaging (2017) 98(9):651–9. doi: 10.1016/j.diii.2017.04.008 28579522

[175] Xing R Gao J Cui Q Wang Q. Strategies to improve the antitumor effect of immunotherapy for hepatocellular carcinoma. Front Immunol (2021) 12:783236. doi: 10.3389/fimmu.2021.783236 34899747 PMC8660685

[176] Harari A Graciotti M Bassani-Sternberg M Kandalaft LE. Antitumour dendritic cell vaccination in a priming and boosting approach. Nat Rev Drug Discovery (2020) 19(9):635–52. doi: 10.1038/s41573-020-0074-8 32764681

[177] Xiao Y Zhang T Ma X Yang QC Yang LL Yang SC. Microenvironment-responsive prodrug-induced pyroptosis boosts cancer immunotherapy. Adv Sci (Weinh) (2021) 8(24):e2101840. doi: 10.1002/advs.202101840 34705343 PMC8693073

[178] Sade-Feldman M Jiao YJ Chen JH Rooney MS Barzily-Rokni M Eliane JP. Resistance to checkpoint blockade therapy through inactivation of antigen presentation. Nat Commun (2017) 8(1):1136. doi: 10.1038/s41467-017-01062-w 29070816 PMC5656607

[179] Gomez S Tabernacki T Kobyra J Roberts P Chiappinelli KB. Combining epigenetic and immune therapy to overcome cancer resistance. Semin Cancer Biol (2020) 65:99–113. doi: 10.1016/j.semcancer.2019.12.019 31877341 PMC7308208

[180] Falkenberg KJ Johnstone RW. Histone deacetylases and their inhibitors in cancer, neurological diseases and immune disorders. Nat Rev Drug Discovery (2014) 13(9):673–91. doi: 10.1038/nrd4360 25131830

[181] Siebenkas C Chiappinelli KB Guzzetta AA Sharma A Jeschke J Vatapalli R. Inhibiting DNA methylation activates cancer testis antigens and expression of the antigen processing and presentation machinery in colon and ovarian cancer cells. PloS One (2017) 12(6):e0179501. doi: 10.1371/journal.pone.0179501 28622390 PMC5473589

[182] Suraweera A O'Byrne KJ Richard DJ. Combination therapy with histone deacetylase inhibitors (Hdaci) for the treatment of cancer: Achieving the full therapeutic potential of hdaci. Front Oncol (2018) 8:92. doi: 10.3389/fonc.2018.00092 29651407 PMC5884928

[183] Zheng H Zhao W Yan C Watson CC Massengill M Xie M. Hdac inhibitors enhance T-cell chemokine expression and augment response to pd-1 immunotherapy in lung adenocarcinoma. Clin Cancer Res (2016) 22(16):4119–32. doi: 10.1158/1078-0432.CCR-15-2584 PMC4987196 26964571

[184] Chen X Pan X Zhang W Guo H Cheng S He Q. Epigenetic strategies synergize with pd-L1/Pd-1 targeted cancer immunotherapies to enhance antitumor responses. Acta Pharm Sin B (2020) 10(5):723–33. doi: 10.1016/j.apsb.2019.09.006 PMC7276686 32528824

[185] Cornel AM Mimpen IL Nierkens S. Mhc class I downregulation in cancer: Underlying mechanisms and potential targets for cancer immunotherapy. Cancers (Basel) (2020) 12(7):1760. doi: 10.3390/cancers12071760 PMC7409324 32630675

[186] Wculek SK Cueto FJ Mujal AM Melero I Krummel MF Sancho D. Dendritic cells in cancer immunology and immunotherapy. Nat Rev Immunol (2020) 20(1):7–24. doi: 10.1038/s41577-019-0210-z 31467405

[187] Ni K Luo T Lan G Culbert A Song Y Wu T. A nanoscale metal-organic framework to mediate photodynamic therapy and deliver cpg oligodeoxynucleotides to enhance antigen presentation and cancer immunotherapy. Angew Chem Int Ed Engl (2020) 59(3):1108–12. doi: 10.1002/anie.201911429 PMC8253508 31642163

[188] Garg AD Coulie PG Van den Eynde BJ Agostinis P. Integrating next-generation dendritic cell vaccines into the current cancer immunotherapy landscape. Trends Immunol (2017) 38(8):577–93. doi: 10.1016/j.it.2017.05.006 28610825

[189] Huang Y Kim BYS Chan CK Hahn SM Weissman IL Jiang W. Improving immune-vascular crosstalk for cancer immunotherapy. Nat Rev Immunol (2018) 18(3):195–203. doi: 10.1038/nri.2017.145 29332937 PMC5922422

[190] Lin YX Wang Y Ding J Jiang A Wang J Yu M. Reactivation of the tumor suppressor pten by mrna nanoparticles enhances antitumor immunity in preclinical models. Sci Transl Med (2021) 13(599):eaba9772. doi: 10.1126/scitranslmed.aba9772 34162754 PMC8284983

[191] Liu YT Sun ZJ. Turning cold tumors into hot tumors by improving T-cell infiltration. Theranostics (2021) 11(11):5365–86. doi: 10.7150/thno.58390 PMC8039952 33859752

[192] Wang L Gao Y Zhang G Li D Wang Z Zhang J. Enhancing Kdm5a and tlr activity improves the response to immune checkpoint blockade. Sci Transl Med (2020) 12(560):eaax2282. doi: 10.1126/scitranslmed.aax2282 32908002

[193] Li X Xiang Y Li F Yin C Li B Ke X. Wnt/Beta-catenin signaling pathway regulating T cell-inflammation in the tumor microenvironment. Front Immunol (2019) 10:2293. doi: 10.3389/fimmu.2019.02293 31616443 PMC6775198

[194] Klempner SJ Bendell JC Villaflor VM Tenner LL Stein SM Rottman JB. Safety, efficacy, and biomarker results from a phase ib study of the anti-Dkk1 antibody dkn-01 in combination with pembrolizumab in advanced esophagogastric cancers. Mol Cancer Ther (2021) 20(11):2240–9. doi: 10.1158/1535-7163.MCT-21-0273 PMC9398109 34482288

[195] Ganesh S Shui X Craig KP Park J Wang W Brown BD. Rnai-mediated beta-catenin inhibition promotes T cell infiltration and antitumor activity in combination with immune checkpoint blockade. Mol Ther (2018) 26(11):2567–79. doi: 10.1016/j.ymthe.2018.09.005 PMC6225018 30274786

[196] Ebert PJR Cheung J Yang Y McNamara E Hong R Moskalenko M. Map kinase inhibition promotes T cell and anti-tumor activity in combination with pd-L1 checkpoint blockade. Immunity (2016) 44(3):609–21. doi: 10.1016/j.immuni.2016.01.024 26944201

[197] Sullivan RJ Hamid O Gonzalez R Infante JR Patel MR Hodi FS. Atezolizumab plus cobimetinib and vemurafenib in braf-mutated melanoma patients. Nat Med (2019) 25(6):929–35. doi: 10.1038/s41591-019-0474-7 31171876

[198] Heckler M Ali LR Clancy-Thompson E Qiang L Ventre KS Lenehan P. Inhibition of Cdk4/6 promotes Cd8 T-cell memory formation. Cancer Discovery (2021) 11(10):2564–81. doi: 10.1158/2159-8290.CD-20-1540 PMC8487897 33941591

[199] Deng J Wang ES Jenkins RW Li S Dries R Yates K. Cdk4/6 inhibition augments antitumor immunity by enhancing T-cell activation. Cancer Discovery (2018) 8(2):216–33. doi: 10.1158/2159-8290.CD-17-0915 PMC5809273 29101163

[200] Zhang QF Li J Jiang K Wang R Ge JL Yang H. Cdk4/6 inhibition promotes immune infiltration in ovarian cancer and synergizes with pd-1 blockade in a b cell-dependent manner. Theranostics (2020) 10(23):10619–33. doi: 10.7150/thno.44871 PMC7482823 32929370

[201] Grauel AL Nguyen B Ruddy D Laszewski T Schwartz S Chang J. Tgfbeta-blockade uncovers stromal plasticity in tumors by revealing the existence of a subset of interferon-licensed fibroblasts. Nat Commun (2020) 11(1):6315. doi: 10.1038/s41467-020-19920-5 33298926 PMC7725805

[202] Chen X Wang L Li P Song M Qin G Gao Q. Dual tgf-beta and pd-1 blockade synergistically enhances mage-A3-Specific Cd8(+) T cell response in esophageal squamous cell carcinoma. Int J Cancer (2018) 143(10):2561–74. doi: 10.1002/ijc.31730 29981155

[203] Batlle E Massague J. Transforming growth factor-beta signaling in immunity and cancer. Immunity (2019) 50(4):924–40. doi: 10.1016/j.immuni.2019.03.024 PMC7507121 30995507

[204] Lind H Gameiro SR Jochems C Donahue RN Strauss J Gulley JM. Dual targeting of tgf-beta and pd-L1 *Via* a bifunctional anti-Pd-L1/Tgf-Betarii agent: Status of preclinical and clinical advances. J Immunother Cancer (2020) 8(1):e000433. doi: 10.1136/jitc-2019-000433 32079617 PMC7057416

[205] Huinen ZR Huijbers EJM van Beijnum JR Nowak-Sliwinska P Griffioen AW. Anti-angiogenic agents - overcoming tumour endothelial cell anergy and improving immunotherapy outcomes. Nat Rev Clin Oncol (2021) 18(8):527–40. doi: 10.1038/s41571-021-00496-y 33833434

[206] Zhao Y Ting KK Li J Cogger VC Chen J Johansson-Percival A. Targeting vascular endothelial-cadherin in tumor-associated blood vessels promotes T-Cell-Mediated immunotherapy. Cancer Res (2017) 77(16):4434–47. doi: 10.1158/0008-5472.CAN-16-3129 28655790

[207] Fukumura D Kloepper J Amoozgar Z Duda DG Jain RK. Enhancing cancer immunotherapy using antiangiogenics: Opportunities and challenges. Nat Rev Clin Oncol (2018) 15(5):325–40. doi: 10.1038/nrclinonc.2018.29 PMC5921900 29508855

[208] Arina A Beckett M Fernandez C Zheng W Pitroda S Chmura SJ. Tumor-reprogrammed resident T cells resist radiation to control tumors. Nat Commun (2019) 10(1):3959. doi: 10.1038/s41467-019-11906-2 31477729 PMC6718618

[209] Tang H Xu X Chen Y Xin H Wan T Li B. Reprogramming the tumor microenvironment through second-near-Infrared-Window photothermal genome editing of pd-L1 mediated by supramolecular gold nanorods for enhanced cancer immunotherapy. Adv Mater (2021) 33(12):e2006003. doi: 10.1002/adma.202006003 33538047

[210] Depil S Duchateau P Grupp SA Mufti G Poirot L. 'Off-the-Shelf' allogeneic car T cells: Development and challenges. Nat Rev Drug Discovery (2020) 19(3):185–99. doi: 10.1038/s41573-019-0051-2 31900462

[211] Gumber D Wang LD. Improving car-T immunotherapy: Overcoming the challenges of T cell exhaustion. EBioMedicine (2022) 77:103941. doi: 10.1016/j.ebiom.2022.103941 35301179 PMC8927848

[212] Song W Zhang M. Use of car-T cell therapy, pd-1 blockade, and their combination for the treatment of hematological malignancies. Clin Immunol (2020) 214:108382. doi: 10.1016/j.clim.2020.108382 32169439

[213] John LB Devaud C Duong CPM Yong CS Beavis PA Haynes NM. Anti-Pd-1 antibody therapy potently enhances the eradication of established tumors by gene-modified T cells. Clin Cancer Res (2013) 19(20):5636–46. doi: 10.1158/1078-0432.Ccr-13-0458 23873688

[214] Gray KD McCloskey JE Vedvyas Y Kalloo OR Eshaky SE Yang Y. Pd1 blockade enhances Icam1-directed car T therapeutic efficacy in advanced thyroid cancer. Clin Cancer Res (2020) 26(22):6003–16. doi: 10.1158/1078-0432.Ccr-20-1523 PMC7709864 32887724

[215] Cherkassky L Morello A Villena-Vargas J Feng Y Dimitrov DS Jones DR. Human car T cells with cell-intrinsic pd-1 checkpoint blockade resist tumor-mediated inhibition. J Clin Invest (2016) 126(8):3130–44. doi: 10.1172/jci83092 PMC4966328 27454297

[216] Wang C Shi F Liu Y Zhang Y Dong L Li X. Anti-Pd-1 antibodies as a salvage therapy for patients with diffuse Large b cell lymphoma who Progressed/Relapsed after Cart19/20 therapy. J Hematol Oncol (2021) 14(1):106. doi: 10.1186/s13045-021-01120-3 34225766 PMC8259370

[217] Chong EA Alanio C Svoboda J Nasta SD Landsburg DJ Lacey SF. Pembrolizumab for b-cell lymphomas relapsing after or refractory to Cd19-directed car T-cell therapy. Blood (2022) 139(7):1026–38. doi: 10.1182/blood.2021012634 PMC9211527 34496014

[218] Wang Z Li N Feng K Chen M Zhang Y Liu Y. Phase I study of car-T cells with pd-1 and tcr disruption in mesothelin-positive solid tumors. Cell Mol Immunol (2021) 18(9):2188–98. doi: 10.1038/s41423-021-00749-x PMC8429583 34381179

[219] Osborne W Chen R Jonnaert M Khokhar NZ Peddareddigari VGR Pule M. Phase 1/2 study of Auto3 the first bicistronic chimeric antigen receptor (Car) targeting Cd19 and Cd22 followed by an anti-Pd1 in patients with Relapsed/Refractory (R/R) diffuse Large b cell lymphoma (Dlbcl): Results of cohort 1 and 2 of the Alexander study. Blood (2019) 134(Supplement_1):246. doi: 10.1182/blood-2019-122724

[220] Hirayama AV Gauthier J Hay KA Sheih A Turtle CJ. Efficacy and toxicity of Jcar014 in combination with durvalumab for the treatment of patients with Relapsed/Refractory aggressive b-cell non-Hodgkin lymphoma. Blood (2018) 132(Suppl_1):1680. doi: 10.1182/blood-2018-99-116745

[221] Zurko J Chaney K Astle JM Johnson BD Hari P Shah NN. Pd-1 blockade after bispecific Lv20.19 car T modulates car T-cell immunophenotype without meaningful clinical response. Haematologica. (2021) 106(10):2788–90. doi: 10.3324/haematol.2021.278461 PMC8485669 33853295

[222] Thommen DS Schumacher TN. T Cell dysfunction in cancer. Cancer Cell (2018) 33(4):547–62. doi: 10.1016/j.ccell.2018.03.012 PMC7116508 29634943

[223] Blank CU Haining WN Held W Hogan PG Kallies A Lugli E. Defining 'T cell exhaustion'. Nat Rev Immunol (2019) 19(11):665–74. doi: 10.1038/s41577-019-0221-9 PMC7286441 31570879

[224] Zarour HM. Reversing T-cell dysfunction and exhaustion in cancer. Clin Cancer Res (2016) 22(8):1856–64. doi: 10.1158/1078-0432.Ccr-15-1849 PMC4872712 27084739

[225] Khan O Giles JR McDonald S Manne S Ngiow SF Patel KP. Tox transcriptionally and epigenetically programs Cd8(+) T cell exhaustion. Nature (2019) 571(7764):211–8. doi: 10.1038/s41586-019-1325-x PMC6713202 31207603

[226] Wherry EJ Kurachi M. Molecular and cellular insights into T cell exhaustion. Nat Rev Immunol (2015) 15(8):486–99. doi: 10.1038/nri3862 PMC4889009 26205583

[227] Tabana Y Moon TC Siraki A Elahi S Barakat K. Reversing T-cell exhaustion in immunotherapy: A review on current approaches and limitations. Expert Opin Ther Targets (2021) 25(5):347–63. doi: 10.1080/14728222.2021.1937123 34056985

[228] Schoffski P Tan DSW Martin M Ochoa-de-Olza M Sarantopoulos J Carvajal RD. Phase I/Ii study of the lag-3 inhibitor ieramilimab (Lag525) +/- anti-Pd-1 spartalizumab (Pdr001) in patients with advanced malignancies. J Immunother Cancer (2022) 10(2):e003776. doi: 10.1136/jitc-2021-003776 35217575 PMC8883259

[229] Curigliano G Gelderblom H Mach N Doi T Tai D Forde PM. Phase I/Ib clinical trial of sabatolimab, an anti-Tim-3 antibody, alone and in combination with spartalizumab, an anti-Pd-1 antibody, in advanced solid tumors. Clin Cancer Res (2021) 27(13):3620–9. doi: 10.1158/1078-0432.CCR-20-4746 33883177

[230] Mettu NB Ulahannan SV Bendell JC Garrido-Laguna I Strickler JH Moore KN. A phase 1a/B open-label, dose-escalation study of etigilimab alone or in combination with nivolumab in patients with locally advanced or metastatic solid tumors. Clin Cancer Res (2022) 28(5):882–92. doi: 10.1158/1078-0432.CCR-21-2780 34844977

[231] Benzon B Zhao SG Haffner MC Takhar M Erho N Yousefi K. Correlation of B7-H3 with androgen receptor, immune pathways and poor outcome in prostate cancer: An expression-based analysis. Prostate Cancer Prostatic Dis (2017) 20(1):28–35. doi: 10.1038/pcan.2016.49 27801901 PMC6512966

[232] Wei H Zhao L Hellstrom I Hellstrom KE Guo Y. Dual targeting of Cd137 Co-stimulatory and pd-1 Co-inhibitory molecules for ovarian cancer immunotherapy. Oncoimmunology (2014) 3:e28248. doi: 10.4161/onci.28248 25050196 PMC4063147

[233] Pauken KE Sammons MA Odorizzi PM Manne S Godec J Khan O. Epigenetic stability of exhausted T cells limits durability of reinvigoration by pd-1 blockade. Science (2016) 354(6316):1160–5. doi: 10.1126/science.aaf2807 PMC5484795 27789795

[234] Wang X He Q Shen H Xia A Tian W Yu W. Tox promotes the exhaustion of antitumor Cd8(+) T cells by preventing Pd1 degradation in hepatocellular carcinoma. J Hepatol (2019) 71(4):731–41. doi: 10.1016/j.jhep.2019.05.015 31173813

[235] Qorraj M Bruns H Bottcher M Weigand L Saul D Mackensen A. The pd-1/Pd-L1 axis contributes to immune metabolic dysfunctions of monocytes in chronic lymphocytic leukemia. Leukemia (2017) 31(2):470–8. doi: 10.1038/leu.2016.214 27479178

[236] Ho PC Bihuniak JD Macintyre AN Staron M Liu X Amezquita R. Phosphoenolpyruvate is a metabolic checkpoint of anti-tumor T cell responses. Cell (2015) 162(6):1217–28. doi: 10.1016/j.cell.2015.08.012 PMC4567953 26321681

[237] Scharping NE Menk AV Whetstone RD Zeng X Delgoffe GM. Efficacy of pd-1 blockade is potentiated by metformin-induced reduction of tumor hypoxia. Cancer Immunol Res (2017) 5(1):9–16. doi: 10.1158/2326-6066.CIR-16-0103 27941003 PMC5340074

[238] Ohue Y Nishikawa H. (Treg) cells in cancer: Can treg cells be a new therapeutic target? Cancer Sci (2019) 110(7):2080–9. doi: 10.1111/cas.14069 PMC6609813 31102428

[239] Whiteside TL. The role of regulatory T cells in cancer immunology. Immunotargets Ther (2015) 4:159–71. doi: 10.2147/ITT.S55415 PMC4918255 27471721

[240] Amoozgar Z Kloepper J Ren J Tay RE Kazer SW Kiner E. Targeting treg cells with gitr activation alleviates resistance to immunotherapy in murine glioblastomas. Nat Commun (2021) 12(1):2582. doi: 10.1038/s41467-021-22885-8 33976133 PMC8113440

[241] Hung AL Maxwell R Theodros D Belcaid Z Mathios D Luksik AS. Tigit and pd-1 dual checkpoint blockade enhances antitumor immunity and survival in gbm. Oncoimmunology (2018) 7(8):e1466769. doi: 10.1080/2162402X.2018.1466769 30221069 PMC6136875

[242] Liu J Yuan Y Chen W Putra J Suriawinata AA Schenk AD. Immune-checkpoint proteins vista and pd-1 nonredundantly regulate murine T-cell responses. Proc Natl Acad Sci U.S.A. (2015) 112(21):6682–7. doi: 10.1073/pnas.1420370112 PMC4450438 25964334

[243] Jacobs JF Punt CJ Lesterhuis WJ Sutmuller RP Brouwer HM Scharenborg NM. Dendritic cell vaccination in combination with anti-Cd25 monoclonal antibody treatment: A phase I/Ii study in metastatic melanoma patients. Clin Cancer Res (2010) 16(20):5067–78. doi: 10.1158/1078-0432.CCR-10-1757 20736326

[244] Santoni M Romagnoli E Saladino T Foghini L Guarino S Capponi M. Triple negative breast cancer: Key role of tumor-associated macrophages in regulating the activity of anti-Pd-1/Pd-L1 agents. Biochim Biophys Acta Rev Cancer (2018) 1869(1):78–84. doi: 10.1016/j.bbcan.2017.10.007 29126881

[245] Gordon SR Maute RL Dulken BW Hutter G George BM McCracken MN. Pd-1 expression by tumour-associated macrophages inhibits phagocytosis and tumour immunity. Nature (2017) 545(7655):495–9. doi: 10.1038/nature22396 PMC5931375 28514441

[246] Eisinger S Sarhan D Boura VF Ibarlucea-Benitez I Tyystjarvi S Oliynyk G. Targeting a scavenger receptor on tumor-associated macrophages activates tumor cell killing by natural killer cells. Proc Natl Acad Sci U.S.A. (2020) 117(50):32005–16. doi: 10.1073/pnas.2015343117 PMC7750482 33229588

[247] Li H Xiao Y Li Q Yao J Yuan X Zhang Y. The allergy mediator histamine confers resistance to immunotherapy in cancer patients *Via* activation of the macrophage histamine receptor H1. Cancer Cell (2022) 40(1):36–52.e9. doi: 10.1016/j.ccell.2021.11.002 34822775 PMC8779329

[248] Grauers Wiktorin H Nilsson MS Kiffin R Sander FE Lenox B Rydstrom A. Histamine targets myeloid-derived suppressor cells and improves the anti-tumor efficacy of pd-1/Pd-L1 checkpoint blockade. Cancer Immunol Immunother (2019) 68(2):163–74. doi: 10.1007/s00262-018-2253-6 PMC6394491 30315349

[249] Loeuillard E Yang J Buckarma E Wang J Liu Y Conboy C. Targeting tumor-associated macrophages and granulocytic myeloid-derived suppressor cells augments pd-1 blockade in cholangiocarcinoma. J Clin Invest (2020) 130(10):5380–96. doi: 10.1172/JCI137110 PMC7524481 32663198

[250] Freeman P Mielgo A. Cancer-associated fibroblast mediated inhibition of Cd8+ cytotoxic T cell accumulation in tumours: Mechanisms and therapeutic opportunities. Cancers (Basel) (2020) 12(9):2687. doi: 10.3390/cancers12092687 PMC7564636 32967079

[251] Narra K Mullins SR Lee HO Strzemkowski-Brun B Magalong K Christiansen VJ. Phase ii trial of single agent Val-boropro (Talabostat) inhibiting fibroblast activation protein in patients with metastatic colorectal cancer. Cancer Biol Ther (2007) 6(11):1691–9. doi: 10.4161/cbt.6.11.4874 18032930

[252] Kieffer Y Hocine HR Gentric G Pelon F Bernard C Bourachot B. Single-cell analysis reveals fibroblast clusters linked to immunotherapy resistance in cancer. Cancer Discovery (2020) 10(9):1330–51. doi: 10.1158/2159-8290.CD-19-1384 32434947

[253] Feig C Jones JO Kraman M Wells RJ Deonarine A Chan DS. Targeting Cxcl12 from fap-expressing carcinoma-associated fibroblasts synergizes with anti-Pd-L1 immunotherapy in pancreatic cancer. Proc Natl Acad Sci U.S.A. (2013) 110(50):20212–7. doi: 10.1073/pnas.1320318110 PMC3864274 24277834

[254] Hanley CJ Mellone M Ford K Thirdborough SM Mellows T Frampton SJ. Targeting the myofibroblastic cancer-associated fibroblast phenotype through inhibition of Nox4. J Natl Cancer Inst (2018) 110(1):10920. doi: 10.1093/jnci/djx121 PMC5903651 28922779

[255] Ford K Hanley CJ Mellone M Szyndralewiez C Heitz F Wiesel P. Nox4 inhibition potentiates immunotherapy by overcoming cancer-associated fibroblast-mediated Cd8 T-cell exclusion from tumors. Cancer Res (2020) 80(9):1846–60. doi: 10.1158/0008-5472.CAN-19-3158 PMC7611230 32122909

[256] Deng L Liang H Burnette B Beckett M Darga T Weichselbaum RR. Irradiation and anti-Pd-L1 treatment synergistically promote antitumor immunity in mice. J Clin Invest (2014) 124(2):687–95. doi: 10.1172/JCI67313 PMC3904601 24382348

[257] Mondini M Levy A Meziani L Milliat F Deutsch E. Radiotherapy-immunotherapy combinations - perspectives and challenges. Mol Oncol (2020) 14(7):1529–37. doi: 10.1002/1878-0261.12658 PMC7332212 32112478

[258] Teng F Kong L Meng X Yang J Yu J. Radiotherapy combined with immune checkpoint blockade immunotherapy: Achievements and challenges. Cancer Lett (2015) 365(1):23–9. doi: 10.1016/j.canlet.2015.05.012 25980820

[259] Shui L Yang X Li J Yi C Sun Q Zhu H. Gut microbiome as a potential factor for modulating resistance to cancer immunotherapy. Front Immunol (2019) 10:2989. doi: 10.3389/fimmu.2019.02989 32010123 PMC6978681

[260] Simpson RC Shanahan E Scolyer RA Long GV. Targeting the microbiome to overcome resistance. Cancer Cell (2021) 39(2):151–3. doi: 10.1016/j.ccell.2021.01.016 33561397

[261] Routy B Gopalakrishnan V Daillere R Zitvogel L Wargo JA Kroemer G. The gut microbiota influences anticancer immunosurveillance and general health. Nat Rev Clin Oncol (2018) 15(6):382–96. doi: 10.1038/s41571-018-0006-2 29636538

[262] Derosa L Routy B Fidelle M Iebba V Alla L Pasolli E. Gut bacteria composition drives primary resistance to cancer immunotherapy in renal cell carcinoma patients. Eur Urol (2020) 78(2):195–206. doi: 10.1016/j.eururo.2020.04.044 32376136

[263] Messaoudene M Pidgeon R Richard C Ponce M Diop K Benlaifaoui M. A natural polyphenol exerts antitumor activity and circumvents anti-Pd-1 resistance through effects on the gut microbiota. Cancer Discov (2022) 12(4):1070–87. doi: 10.1158/2159-8290.CD-21-0808 PMC9394387 35031549

[264] Cortes J Cescon DW Rugo HS Nowecki Z Im SA Yusof MM. Pembrolizumab plus chemotherapy versus placebo plus chemotherapy for previously untreated locally recurrent inoperable or metastatic triple-negative breast cancer (Keynote-355): A randomised, placebo-controlled, double-blind, phase 3 clinical trial. Lancet (2020) 396(10265):1817–28. doi: 10.1016/S0140-6736(20)32531-9 33278935

[265] Sun JM Shen L Shah MA Enzinger P Adenis A Doi T. Pembrolizumab plus chemotherapy versus chemotherapy alone for first-line treatment of advanced oesophageal cancer (Keynote-590): A randomised, placebo-controlled, phase 3 study. Lancet (2021) 398(10302):759–71. doi: 10.1016/S0140-6736(21)01234-4 34454674

[266] Chung HC Bang YJ S Fuchs C Qin SK Satoh T Shitara K. First-line Pembrolizumab/Placebo plus trastuzumab and chemotherapy in Her2-positive advanced gastric cancer: Keynote-811. Future Oncol (2021) 17(5):491–501. doi: 10.2217/fon-2020-0737 33167735 PMC8411394

[267] Paz-Ares L Dvorkin M Chen Y Reinmuth N Hotta K Trukhin D. Durvalumab plus platinum-etoposide versus platinum-etoposide in first-line treatment of extensive-stage small-cell lung cancer (Caspian): A randomised, controlled, open-label, phase 3 trial. Lancet (2019) 394(10212):1929–39. doi: 10.1016/S0140-6736(19)32222-6 31590988

[268] Theelen W Peulen HMU Lalezari F van der Noort V de Vries JF Aerts J. Effect of pembrolizumab after stereotactic body radiotherapy vs pembrolizumab alone on tumor response in patients with advanced non-small cell lung cancer: Results of the pembro-rt phase 2 randomized clinical trial. JAMA Oncol (2019) 5(9):1276–82. doi: 10.1001/jamaoncol.2019.1478 PMC6624814 31294749

[269] McBride S Sherman E Tsai CJ Baxi S Aghalar J Eng J. Randomized phase ii trial of nivolumab with stereotactic body radiotherapy versus nivolumab alone in metastatic head and neck squamous cell carcinoma. J Clin Oncol (2021) 39(1):30–7. doi: 10.1200/JCO.20.00290 PMC8462641 32822275

[270] Hodi FS Chesney J Pavlick AC Robert C Grossmann KF McDermott DF. Combined nivolumab and ipilimumab versus ipilimumab alone in patients with advanced melanoma: 2-year overall survival outcomes in a multicentre, randomised, controlled, phase 2 trial. Lancet Oncol (2016) 17(11):1558–68. doi: 10.1016/S1470-2045(16)30366-7 PMC5630525 27622997

[271] Tawbi HA Schadendorf D Lipson EJ Ascierto PA Matamala L Castillo Gutierrez E. Relatlimab and nivolumab versus nivolumab in untreated advanced melanoma. N Engl J Med (2022) 386(1):24–34. doi: 10.1056/NEJMoa2109970 34986285 PMC9844513

[272] Goldman JW Dvorkin M Chen Y Reinmuth N Hotta K Trukhin D. Durvalumab, with or without tremelimumab, plus platinum-etoposide versus platinum-etoposide alone in first-line treatment of extensive-stage small-cell lung cancer (Caspian): Updated results from a randomised, controlled, open-label, phase 3 trial. Lancet Oncol (2021) 22(1):51–65. doi: 10.1016/S1470-2045(20)30539-8 33285097

[273] Kelly RJ Lee J Bang YJ Almhanna K Blum-Murphy M Catenacci DVT. Safety and efficacy of durvalumab and tremelimumab alone or in combination in patients with advanced gastric and gastroesophageal junction adenocarcinoma. Clin Cancer Res (2020) 26(4):846–54. doi: 10.1158/1078-0432.CCR-19-2443 PMC7748730 31676670

[274] Rizvi NA Cho BC Reinmuth N Lee KH Luft A Ahn MJ. Durvalumab with or without tremelimumab vs standard chemotherapy in first-line treatment of metastatic non-small cell lung cancer: The mystic phase 3 randomized clinical trial. JAMA Oncol (2020) 6(5):661–74. doi: 10.1001/jamaoncol.2020.0237 PMC7146551 32271377

[275] Ferris RL Haddad R Even C Tahara M Dvorkin M Ciuleanu TE. Durvalumab with or without tremelimumab in patients with recurrent or metastatic head and neck squamous cell carcinoma: Eagle, a randomized, open-label phase iii study. Ann Oncol (2020) 31(7):942–50. doi: 10.1016/j.annonc.2020.04.001 32294530

[276] Postow MA Sidlow R Hellmann MD. Immune-related adverse events associated with immune checkpoint blockade. N Engl J Med (2018) 378(2):158–68. doi: 10.1056/NEJMra1703481 29320654

[277] Horvath L Pircher A. Asco 2020 non-small lung cancer (Nsclc) personal highlights. Memo (2021) 14(1):66–9. doi: 10.1007/s12254-020-00673-2 33456617 PMC7804575

[278] Nocera L Karakiewicz PI Wenzel M Tian Z Shariat SF Saad F. Clinical outcomes and adverse events after first-line treatment in metastatic renal cell carcinoma: A systematic review and network meta-analysis. J Urol. (2022) 207(1):16–24. doi: 10.1097/JU.0000000000002252 34546767

[279] Powles T Plimack ER Soulieres D Waddell T Stus V Gafanov R. Pembrolizumab plus axitinib versus sunitinib monotherapy as first-line treatment of advanced renal cell carcinoma (Keynote-426): Extended follow-up from a randomised, open-label, phase 3 trial. Lancet Oncol (2020) 21(12):1563–73. doi: 10.1016/S1470-2045(20)30436-8 33284113

[280] Reck M Mok TSK Nishio M Jotte RM Cappuzzo F Orlandi F. Atezolizumab plus bevacizumab and chemotherapy in non-Small-Cell lung cancer (Impower150): Key subgroup analyses of patients with egfr mutations or baseline liver metastases in a randomised, open-label phase 3 trial. Lancet Respir Med (2019) 7(5):387–401. doi: 10.1016/S2213-2600(19)30084-0 30922878

[281] Galle PR Finn RS Qin S Ikeda M Zhu AX Kim TY. Patient-reported outcomes with atezolizumab plus bevacizumab versus sorafenib in patients with unresectable hepatocellular carcinoma (Imbrave150): An open-label, randomised, phase 3 trial. Lancet Oncol (2021) 22(7):991–1001. doi: 10.1016/S1470-2045(21)00151-0 34051880

[282] Motzer RJ Robbins PB Powles T Albiges L Haanen JB Larkin J. Avelumab plus axitinib versus sunitinib in advanced renal cell carcinoma: Biomarker analysis of the phase 3 javelin renal 101 trial. Nat Med (2020) 26(11):1733–41. doi: 10.1038/s41591-020-1044-8 PMC8493486 32895571

[283] Choueiri TK Powles T Burotto M Escudier B Bourlon MT Zurawski B. Nivolumab plus cabozantinib versus sunitinib for advanced renal-cell carcinoma. N Engl J Med (2021) 384(9):829–41. doi: 10.1056/NEJMoa2026982 PMC8436591 33657295

[284] Xu Q Wang J Sun Y Lin Y Liu J Zhuo Y. Efficacy and safety of sintilimab plus anlotinib for pd-L1-Positive recurrent or metastatic cervical cancer: A multicenter, single-arm, prospective phase ii trial. J Clin Oncol (2022) 40(16)1795–1805. doi: 10.1200/JCO.21.02091 PMC9148684 35192397

